# Radiogenomic markers enable risk stratification and inference of mutational pathway states in head and neck cancer

**DOI:** 10.1007/s00259-022-05973-9

**Published:** 2022-09-26

**Authors:** Clemens P. Spielvogel, Stefan Stoiber, Laszlo Papp, Denis Krajnc, Marko Grahovac, Elisabeth Gurnhofer, Karolina Trachtova, Vojtech Bystry, Asha Leisser, Bernhard Jank, Julia Schnoell, Lorenz Kadletz, Gregor Heiduschka, Thomas Beyer, Marcus Hacker, Lukas Kenner, Alexander R. Haug

**Affiliations:** 1 Christian Doppler Laboratory for Applied Metabolomics, Vienna, Austria; 2 Department of Biomedical Imaging and Image-Guided Therapy, Division of Nuclear Medicine, Medical University of Vienna, Vienna, Austria; 3 Clinical Institute of Pathology, Medical University of Vienna, Vienna, Austria; 4 Center for Medical Physics and Biomedical Engineering, Medical University of Vienna, Vienna, Austria; 5 Centre for Molecular Medicine, Central European Institute of Technology, Brno, Czech Republic; 6 Department of Otorhinolaryngology, Head and Neck Surgery, Medical University of Vienna, Vienna, Austria

**Keywords:** Head and neck cancer, Biomarkers, Radiomics, Machine learning, Artificial intelligence, Cancer genomics

## Abstract

**Purpose:**

Head and neck squamous cell carcinomas (HNSCCs) are a molecularly, histologically, and clinically heterogeneous set of tumors originating from the mucosal epithelium of the oral cavity, pharynx, and larynx. This heterogeneous nature of HNSCC is one of the main contributing factors to the lack of prognostic markers for personalized treatment. The aim of this study was to develop and identify multi-omics markers capable of improved risk stratification in this highly heterogeneous patient population.

**Methods:**

In this retrospective study, we approached this issue by establishing radiogenomics markers to identify high-risk individuals in a cohort of 127 HNSCC patients. Hybrid *in vivo* imaging and whole-exome sequencing were employed to identify quantitative imaging markers as well as genetic markers on pathway-level prognostic in HNSCC. We investigated the deductibility of the prognostic genetic markers using anatomical and metabolic imaging using positron emission tomography combined with computed tomography. Moreover, we used statistical and machine learning modeling to investigate whether a multi-omics approach can be used to derive prognostic markers for HNSCC.

**Results:**

Radiogenomic analysis revealed a significant influence of genetic pathway alterations on imaging markers. A highly prognostic radiogenomic marker based on cellular senescence was identified. Furthermore, the radiogenomic biomarkers designed in this study vastly outperformed the prognostic value of markers derived from genetics and imaging alone.

**Conclusion:**

Using the identified markers, a clinically meaningful stratification of patients is possible, guiding the identification of high-risk patients and potentially aiding in the development of effective targeted therapies.

**Graphical abstract:**

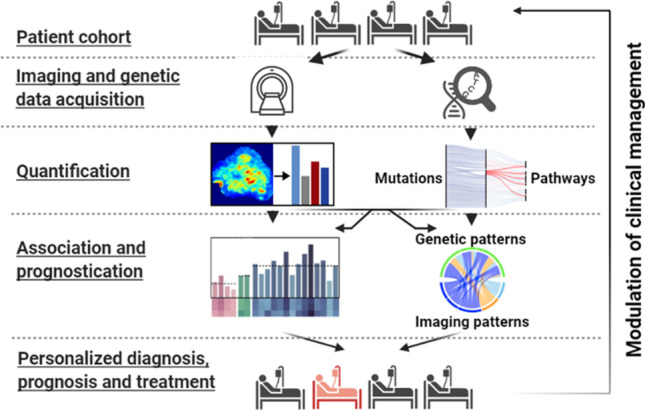

**Supplementary Information:**

The online version contains supplementary material available at 10.1007/s00259-022-05973-9.

## Background

Worldwide, head and neck cancer accounts for more than 430,000 annual deaths and over 830,000 individuals are diagnosed with head and neck cancer every year [1]. Head and neck squamous cell carcinoma (HNSCC) accounts for approximately 90% of all head and neck cancers [2]. HNSCC originates from the epithelial cells outlining the mucosa of various cavities in the head and neck area. The anatomical, clinical, histological, and molecular heterogeneity of HNSCC has been a limiting factor for the development of personalized treatments. Today, PD-L1 expression and human papilloma virus (HPV) infection status are the only considered biomarkers for personalized clinical management of HNSCC patients [3, 4]. Consequently, further markers are urgently needed for the stratification of clinically meaningful groups to better tailor the management of these patients to their individual characteristics.

Metabolic *in vivo* imaging provided by technologies such as positron emission tomography combined with computed tomography (PET/CT) is a non-invasive way to capture information about biological processes on a whole-body scale. *In vivo* imaging further enables the high-throughput acquisition of quantitative imaging features, referred to as radiomics. Radiomics has been deployed to describe tumor characteristics, such as shape and heterogeneity on a quantitative level, which have been shown to deliver prognostic information in various settings [5, 6].

In parallel to the advancements of diagnostic imaging modalities driven by clinical research, mechanistic cancer research has been capitalizing on the revolution in sequencing technologies. Today, genomics provides crucial diagnostic information to advance toward personalized cancer medicine. Tissue-based DNA biomarkers comprise some of the most important prognostic factors in HNSCC [7]. These prognostic markers can be useful for the monitoring and selection of patients for a specific treatment [6, 8]. In contrast to these gene-level markers, pathway-level biomarkers are largely unexplored. Still, since mutations are only one of several ways to inactivate tumor suppressors or activate oncogenes [9], genetic analysis inherently provides an important but only partial view of the cancer phenotype. Radiomic features, on the other hand, have the potential to provide functional information on the activity of oncogenic drivers at a holistic level. Thus, an approach combining the strength of both technologies which is referred to as radiogenomics has the potential to raise currently underexplored synergies to advance the personalized management of cancer patients.

**Fig. 1:**
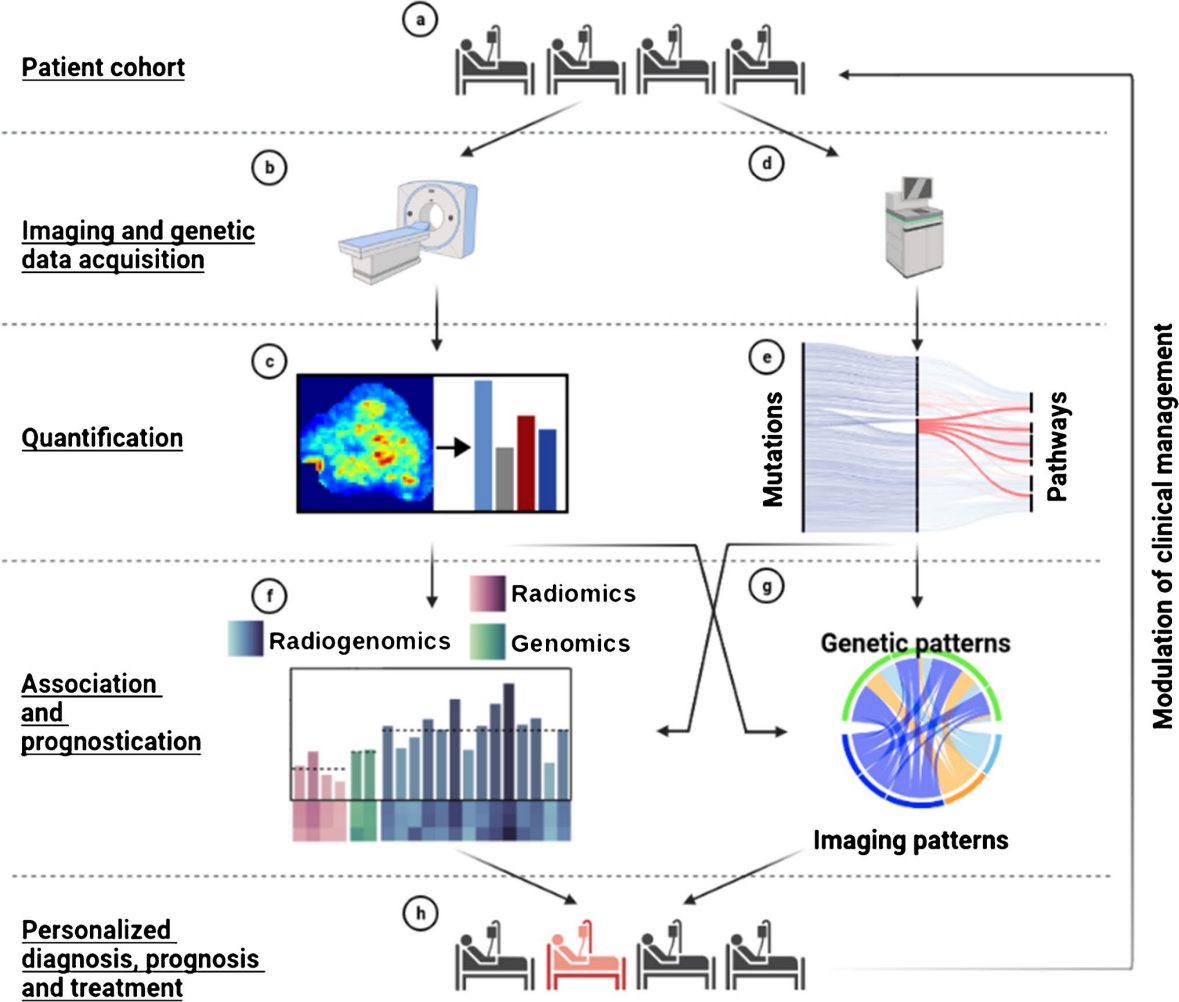
Workflow diagram of the study. **a** Primary tumor tissue from 62 patients with HNSCC was acquired through surgical biopsy. **b** Image data acquisition using [.^18^F]FDG PET/CT. **c** Quantification of tumor characteristics based on imaging data using radiomics. **d** DNA extraction from solid tumor tissue and subsequent whole exome sequencing. **e** Quantification of genetically disrupted pathways related to cell growth and death as well as energy metabolism using combined annotation dependent depletion (CADD) scores [10]. **f** Identification and evaluation of prognostic radiomic, genomic, and radiogenomic features using statistical and machine learning approaches. **g** Statistical assessment of the association and complementary information of pathway-level genetic features and non-redundant radiomic features. **h** Patient-tailored diagnosis, prognosis, and treatment based on the detected radiogenomic markers and associated risk assessment. This figure was created using BioRender (biorender.com)

The aim of the present study was therefore threefold (Fig. 1): (1) the identification of quantitative and prognostic [^18^F]FDG PET/CT imaging and genetic markers in HNSCC; (2) the assessment of the association of previously identified imaging markers with pathways related to cell proliferation and energy metabolism; (3) to investigate if complementary information within imaging and genetic patterns can be used to create combined radiogenomic markers with improved prognostic value over imaging or genetic markers only.

## Materials and methods

### Patient data

One hundred and twenty-seven (127) patients diagnosed with HNSCC between June 8, 2006 and July 31, 2015 with whole-body [^18^F]FDG PET/CT scans at the General Hospital Vienna were retrospectively enrolled into the study. CT was acquired using contrast enhancement with 100 ml Iomeron 400 mg/ml. Overall, 2 patients were excluded due to lesion sizes below 64 voxels [11], 4 due to a second primary tumor, and 59 due to missing or insufficient tumor tissue for DNA extraction, resulting in 62 patients for further analysis. The clinical annotation was acquired by the head-and-neck surgeon taking the tissue biopsies and included overall survival (OS) starting from the date of histologically confirmed diagnosis. An overview of patient characteristics is provided in Table 1. All biopsies originated from histologically confirmed head and neck squamous cell carcinomas. The study was approved by the institutional review board with ethics ID 1649/2016 at the General Hospital of Vienna.

**Table 1:** Characteristics of the 62 patients included for analysis

Patient characteristics	
Median age, years (range)	57 (35–83)
Median overall survival, months (range)	25 (0–130)
Male, n (%)	45 (73)
Female, n (%)	17 (27)
Treatment naive at tissue acquisition, n (%)	52 (84)
Clinical stage, n (%)	
I	4 (6)
II	5 (8)
III	4 (6)
IVA	38 (61)
IVB	3 (5)
IVC	7 (11)
Not reported	1 (2)
Localization, n (%)	
Oral cavity	35 (56)
Oropharynx	16 (26)
Hypopharynx	6 (10)
Larynx	4 (6)
Nasal sinuses	1 (2)

### DNA extraction, whole-exome sequencing, and sequencing data analysis

DNA was extracted from formalin-fixed paraffin-embedded samples and sequenced using whole-exome sequencing (WES). Details on DNA extraction and WES analysis can be found in Supplement section 1 under “DNA extraction and whole exome sequencing.”

#### DNA sequencing analysis

Raw reads were mapped to the genomic reference GRCh38 using the Burrows–Wheeler Alignment (BWA) tool [12]. Small variants were detected using Strelka2 [13] and VarDict [14] variant callers independently, and the resulting variants were merged. Variants were annotated with the Variant Effect Predictor (VEP) tool from Ensemble [15] including the annotation of CADD scores [10, 16, 17]. Resulting annotated variants were joined across the cohort and germline variants were filtered. The discrimination of somatic and germline variants was based on a somatic tumor variant filtering strategy from Sukhai et al. [18], with additional filters added and parameters adjusted in order to minimize the ratio of known germline variants resulting from a set of 15 paired normal tissues. The final somatic variant filtering was performed as follows. Only variants present in less than 10% of samples were kept. Variants called by both Strelka2 and VarDict with a number of variant reads above 10 and variants called by only one variant caller with a number of variant reads above 20 were kept. Three population variant databases were used for variant filtering including 1000 genome [19], Gnomad [20], and the NHLBI Exome Sequencing Project [21]. Variants with a minor allele frequency below 1% for the non-Finnish European group in all three databases were kept. Variants with a record in ClinVar database [22] with significance “benign” or “likely benign” were removed.

#### Pathway-level disruption scores and pathway selection

Mutation-level combined annotation dependent depletion (CADD) scores [10] were summed over all variants in associated genes to derive gene-level CADD scores indicating the functional disruption of each gene. The KEGG pathway database [23] was used to assign genes to corresponding pathways. Pathway CADD scores were computed as sum of gene-level CADD scores for all genes in the respective pathway. Pathways were considered for the analysis if they were either annotated as related to energy metabolism or to cell growth and death based on the KEGG pathway database. Pathways were excluded if they do not exist in humans or were irrelevant for somatic tissue (Supplementary Table 6).

Gene-level CADD score cutoffs were unlikely to be accurately determined by setting a uniform cutoff [24]. Therefore, we used the prognostically relevant cutoffs as determined by the survival analysis for the dichotomization of each pathway’s score individually. By doing so, we derived prognostically relevant binary states, functional or disrupted, for each pathway (Supplementary Fig. 8). The binary pathway states were used for the associated with imaging patterns and the derivation of radiogenomic markers.

#### Delineation

Two board-registered nuclear medicine specialists at the Division of Nuclear Medicine at the Medical University of Vienna performed tumor boundary delineation to derive volumes of interest (VOIs) from the whole-body images. For each patient, one delineation was created based on the agreement of the two nuclear medicine specialists. Delineation of lesions and background tissue were performed utilizing semi-automated iso-count VOI tools from the commercially available Hybrid 3D software version 4.0.0 (Hermes Medical Solutions AB, Stockholm, Sweden). If required, a slice-by-slice modification was performed [25, 26]. Delineation in PET/CT images was guided by the PET image. VOIs were dilated by 5 voxels into every spatial dimension.

#### Radiomic feature extraction and preprocessing

The SUV maps of the VOIs were normalized using a standardized reference region before performing interpolation to 2 and 4 mm. Radiomic features were extracted from the resulting VOIs using an IBSI-conform in-house framework. Overall, 104 Imaging Biomarker Standardization Initiative (IBSI)-conform radiomic features were extracted, 52 from the background-normalized PET and the corresponding CT each. Details on the extraction and preprocessing of image biomarkers are outlined in Supplemental section 1 under “Radiomic feature extraction and preprocessing.”

### Development of radiogenomic markers

Radiogenomic features were created by combining the most prognostic pathways (*p* < 0.05) with the most prognostic radiomic features (*p* < 0.05). Each radiogenomic feature consists of a radiomic–genomic feature pair with one radiomic and one pathway feature. For each radiomic–genomic feature pair, four binary radiogenomic features were created (pathway-disrupted and radiomic-high, pathway-disrupted and radiomic-low, pathway-functional and radiomic-high, pathway-disrupted and radiomic-low). For example, the radiogenomic feature *cellular senescence (functional)-CT ih.kurt (high)* was defined to be “present” for a patient if the patient has a functional cellular senescence pathway and a high value (above threshold determined by survival analysis) for the CT radiomic feature *ih.kurt*. In all other cases, the radiogenomic feature value was defined as “absent.” From the total of 84 radiogenomic markers, only those with sufficiently large subgroups for survival analysis (at least 15% samples in each group) were considered, leaving 49 radiogenomic markers for further analysis.

### Statistical analysis

Survival analysis was conducted using two-sided logrank tests with an optimized cutoff and OS. Logrank tests, two-sided Cox proportional hazard models, and plotting for Kaplan–Meier curves were performed using the lifelines Python package. No survival analysis was performed if one of the groups contained less than 15% samples. The association between radiomic features and pathway-level scores was performed using the non-parametric, two-sided Mann–Whitney *U* test implementation of the SciPy Python package. Bonferroni correction was applied for all statistical analyses to account for multiple testing.

#### Machine learning classification

Binary machine learning (ML) classification models were built using Dedicaid AutoML version 1.1 (Dedicaid GmbH, Vienna, Austria) via a stacked and mixed ensemble approach. Algorithms used in the ensemble included random forest, support vector machine, and a multi-Gaussian genetic algorithm. Preprocessing included standardization of input features and removal of redundant features. In case of label imbalance, oversampling was employed on the training data via the synthetic minority oversampling technique (SMOTE) [27]. A total of 20 genomic, radiomic, and radiogenomic features were included which were identified to be prognostic in the preceding univariate analyses. Prediction target labels were generated by dichotomization of the continuous OS information. Three binary classification models were created for OS greater 24 months, OS greater median (25 months) and OS greater 36 months. Results were validated using 100-fold Monte Carlo cross-validation with a training-to-test sample ratio of 80:20. Details on the ML analysis can be found in Supplement section 2.

#### Feature importance measurement

Feature importance measurement was based on R-squared ranking [28]. R-squared ranks were determined on the binary target labels for each of the ML models, leading to one feature importance ranking per model. The final importance was calculated as the average feature importance across all 100 Monte Carlo cross-validation folds. The importance metrics were further normalized to a sum of 100 (%) per model.

#### Code and visualization tools

All analyses were conducted using Python 3. Packages used included pandas 1.0.3, numpy 1.19.2, and scikit-learn 0.23.2. For the survival analysis and plotting of associated Kaplan–Meier curves, lifelines 0.24.13 was used. For any other statistical analysis, we used SciPy 1.4.1. Visualizations were created using Matplotlib 3.2.1 and Seaborn 0.11.1. For the creation of rain cloud plots, we used the package Ptprince 0.2. For the creation of sankey diagrams, Plotly 4.4.1 was used. The graphical abstract was created using BioRender (biorender.com).

## Results

### Processing and analysis of the radiomic features

**Fig. 2:**
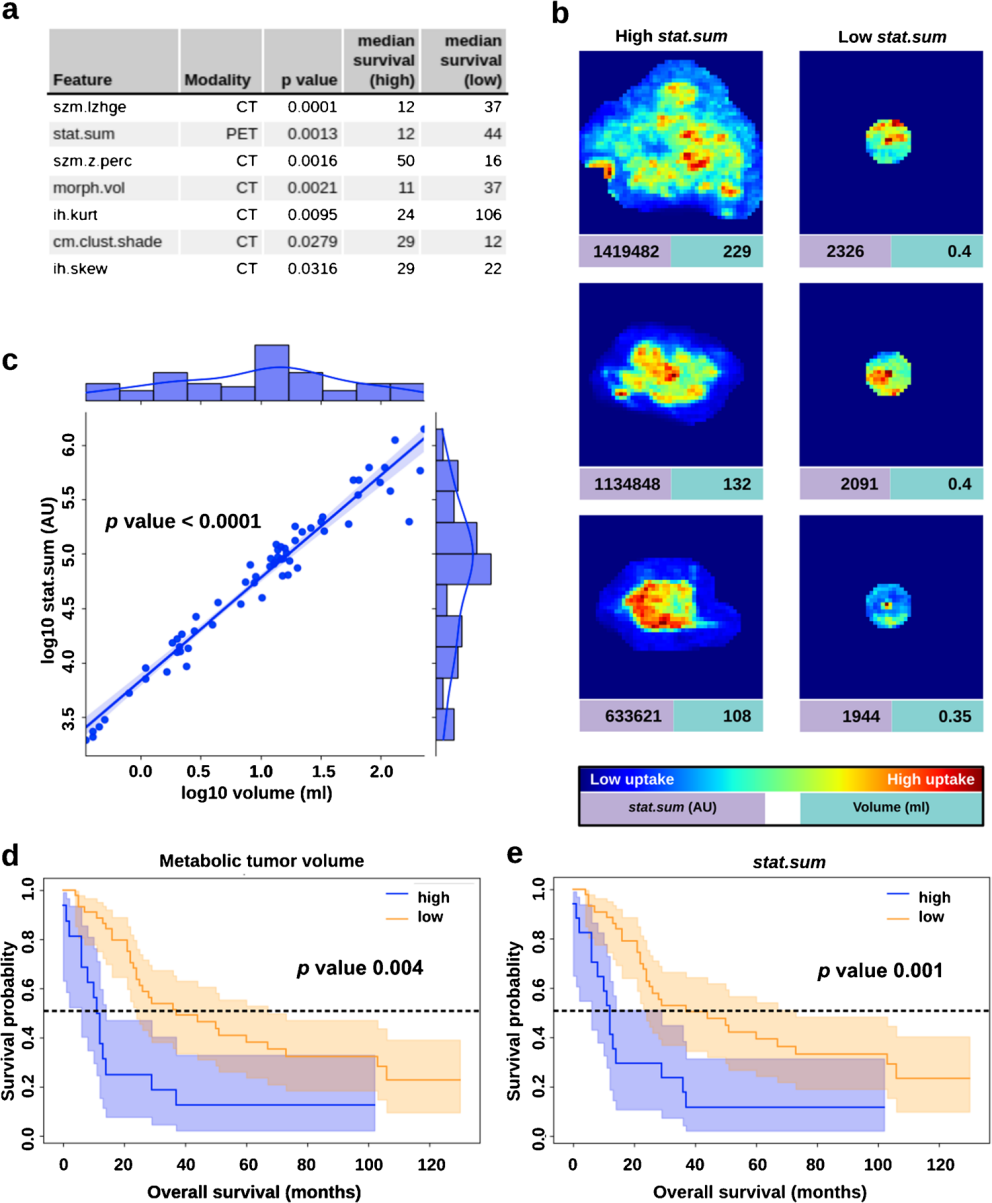
*stat.sum* captures information of metabolic tumor volume (MTV) and improves prognostic stratification. **a** Most prognostic radiomic features (*p* < 0.05) with associated modalities and respective survival analysis results. Ordered by *p* value. **b** Coronal maximum intensity projections (MIPs) of the PET images for the three lesions with the highest *stat.sum* (left column) and lowest *stat.sum* (right column). Red values indicate a high [.^18^F]FDG uptake while blue values indicate a low or no uptake. **c** Pearson correlation between MTV and *stat.sum* after applying log transformation. **d** Kaplan–Meier curve for MTV. **e** Kaplan–Meier curve for *stat.sum*. Radiomic feature values are shown in arbitrary units (AU)

IBSI-conform radiomic features were extracted from [^18^F]FDG PET/CT images of primary lesions from 62 patients with HNSCC [29]. After redundancy removal [26, 30], 4 PET-based and 10 CT-based features remained for further analysis (Supplemental Fig. 1). Independent assessment of PET and CT features identified two texture CT features, *szm.lzhge* (*p* 6.4 * 10^−5^) and *szm.z.perc* (*p* 0.0016), one morphological feature, *morph.vol* (*p* 0.0021), and one intensity-related PET feature, *stat.sum* (*p* 0.0013), to be prognostic (Fig. 2a).

On visual inspection of tumors, lesions with high PET-based *stat.sum* were associated with large volumes (Fig. 2b). Since PET-based metabolic tumor volume (MTV) has been proposed as a prognostic marker for multiple cancers, including HNSCC [31], we further investigated the association between *stat.sum* and MTV. The analysis confirmed a strong correlation (*p* < 0.0001) (Fig. 2c). CT-based morph.vol was the only additional feature correlated with MTV, indicating no systematic effect of volume on the radiomic features. Furthermore, *stat.sum* was associated with a slightly improved prognostic value over MTV (*p* 0.0013 vs. 0.0040) (Fig. 2d, e).

None of the SUV-based features, SUVmax, SUVmin, SUVmean, SUVpeak, and SUV total lesion glycolysis (TLG), were significantly prognostic after Bonferroni correction (*p* < 0.01) (Supplementary Table 1 and Supplementary Figs. 2–6), indicating a higher prognostic value of radiomic features over SUV metrics in this study cohort.

### Processing of genetic data and creation of pathway disruption scores

**Fig. 3:**
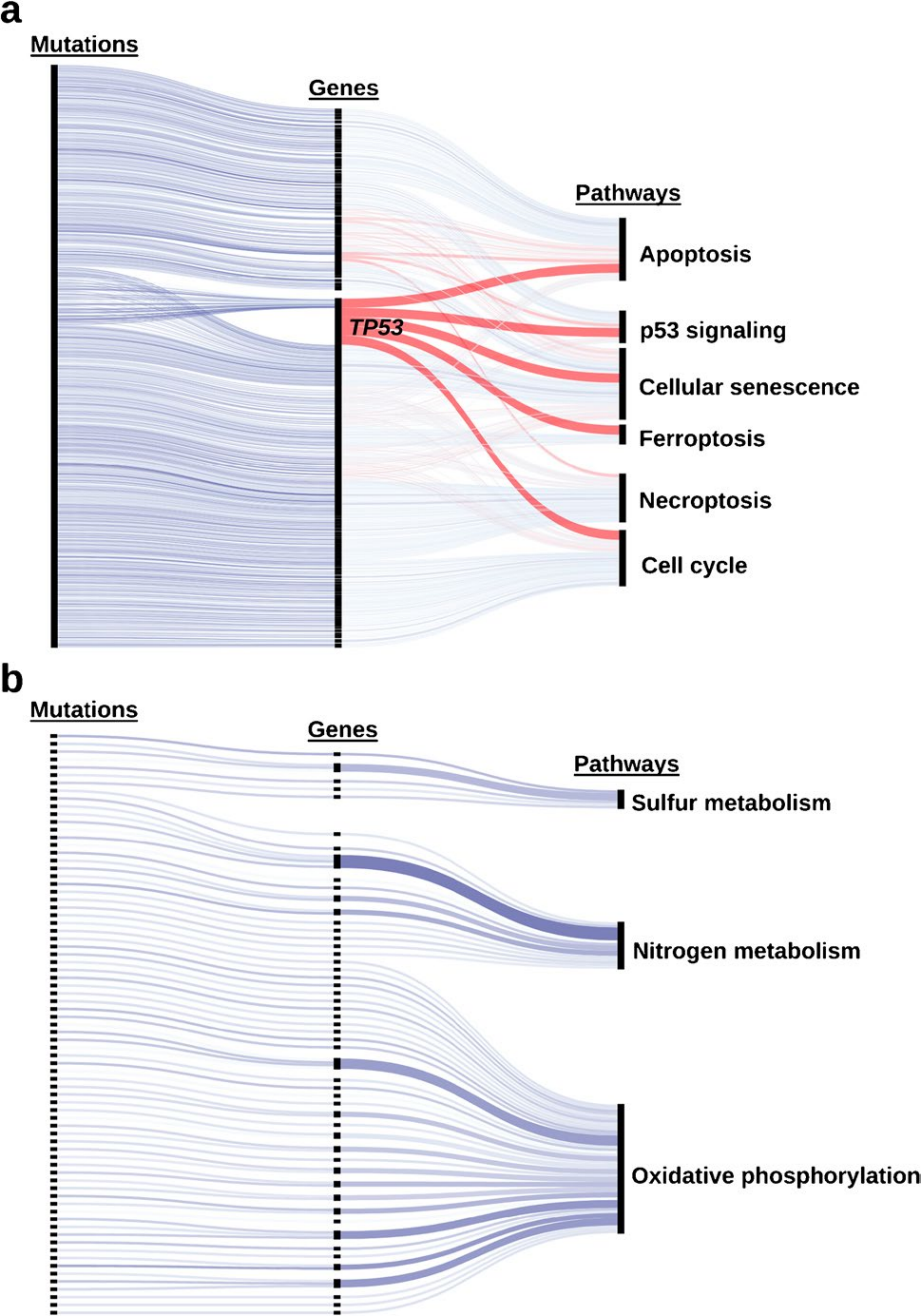
Composition of pathway CADD scores from gene- and mutation-level scores for KEGG pathways associated with cell growth and death (**a**) and energy metabolism (**b**). Links of genes influencing multiple pathways are shown in red. The color intensity of links indicates the CADD score of mutations and sums of CADD scores over all mutations for mutations and genes, respectively. Hence, darker blue or red indicate a higher CADD score (disrupted) while a lighter color indicates a low CADD score (functional). The width of links between genes and pathways indicates the number of mutations in a gene over all patients in our cohort. The fully annotated, interactive version of this figure is available at https://cspielvogel.github.io/cadd-diagram/cadd_composition_cell_growth_and_death.html and https://cspielvogel.github.io/cadd-diagram/cadd_composition_energy_metabolism.html for cell growth and death or energy metabolism, respectively

Solid tissue from primary tumors of 62 patients was acquired and WES was performed. A total of 15,689 mutations in 8502 genes was detected across all patients. The most mutated genes included MUC4 (66%), TTN (35%), TP53 (27%), MUC12 (24%), and CSMD3 (23%). The relation of mutation-, gene-, and pathway-level CADD scores for the six selected cell growth and death-related pathways and three energy metabolism–related pathways is visualized in two interactive CADD score diagrams (representatively shown in Fig. 3). Of the nine pathways, survival analysis identified cellular senescence and apoptosis to be significantly prognostic (*p* < 0.008).

The proliferation-related CADD composition diagram (Fig. 3a) suggested a major role of the *TP53* gene in deriving the pathway-level CADD score for p53 signaling, cellular senescence, ferroptosis, cell cycle, and apoptosis. However, survival analysis revealed that *TP53* alone has no prognostic value (*p* 0.18) (Supplementary Fig. 7). Since mutation frequencies in 8486 of 8502 mutated genes was below 15%, no additional analyses on single gene level were carried out.

### Association of radiomics and pathway disruption scores

**Fig. 4:**
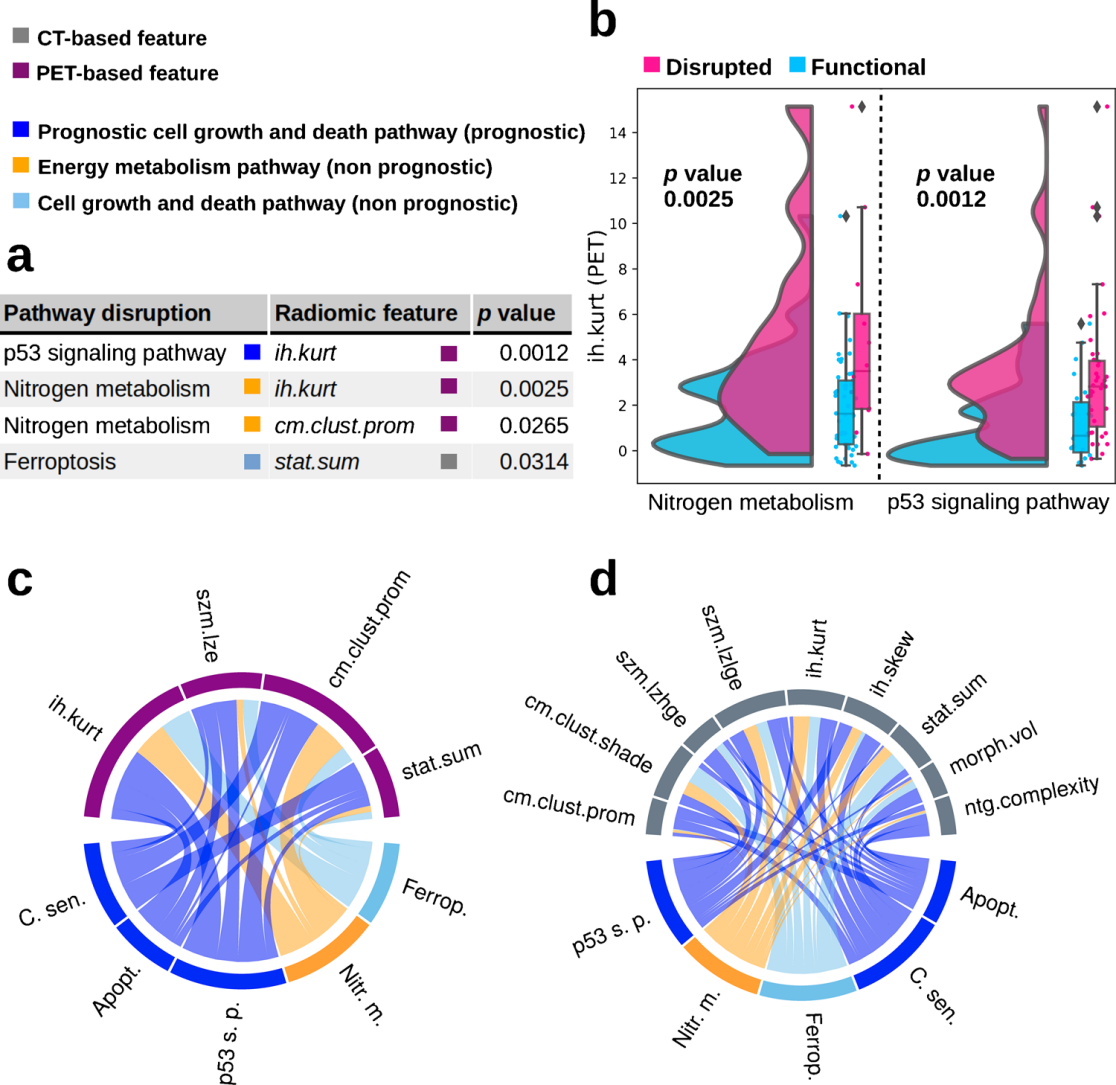
Association of PET- and CT-based radiomic features with pathways related to cell growth and death as well as energy metabolism. **a** Pathways and linked radiomic features with *p* values below 0.05. **b** Distribution of the two most significant associations: PET-based radiomic histogram feature excess discretized intensity kurtosis (*ih.kurt*) depending on the functional state of two pathways, nitrogen metabolism (left) and p53 signaling (right). For each pathway, the distribution of the radiomic feature *ih.kurt* (PET) is visualized for patients with functional (blue) and disrupted (pink) genetic status via kernel density estimation. A higher width of the curve area at a given radiomic feature value on the *y*-axis indicates a higher probability of a patient to have the respective radiomic feature value as estimated by the kernel density estimation. Furthermore, for each pathway, two boxplots indicate the distribution of the radiomic feature for patients with functional and disrupted genetic status. Radiomic features are displayed in arbitrary units. **c** Associations between PET radiomic features and pathways. **d** Associations between CT radiomic features and pathways. The width of the links indicates the inverse *p* value within each of the chord plots. Pathways include cellular senescence (C. sen.), apoptosis (Apopt.), p53 signaling pathway (p53 s. p.), nitrogen metabolism (Nitr. m.), and ferroptosis (Ferrop.)

Significant associations between four radiomic-pathway pairs were identified (*p* < 0.05) (Fig. 4a). A significant association was found between p53 signaling and PET-based *ih.kurt* (*p* < 0.002) (Fig. 4). The overlap of radiomic feature distributions for both functional pathway states identified *ih.kurt* as indicator but not as an error-free predictor of the pathway states (Fig. 4b). Multiple other radiomic-pathway combinations are potentially associated, but did not reach significance (Fig. 4c, d). A full list of association results is shown in Supplementary Table 2.

### Prognostic value of radiogenomic markers

Since the preceding analysis indicated pathway states cannot be predicted solely from imaging markers (Fig. 4b), the incorporation of complementary information via combining radiomic and pathway features to radiogenomic features was investigated. Of the 49 radiogenomic markers (Supplementary Table 3), 14 were significantly prognostic (*p* < 0.001). Seven radiogenomic markers were more prognostic than the most prognostic univariate marker *szm.lzhge* (*p* < 0.0001). The best performing radiogenomic marker was *cellular senescence (functional)-CT ih.kurt (high)* indicating a worse prognosis (*p* 5.5 * 10^−8^).

**Fig. 5:**
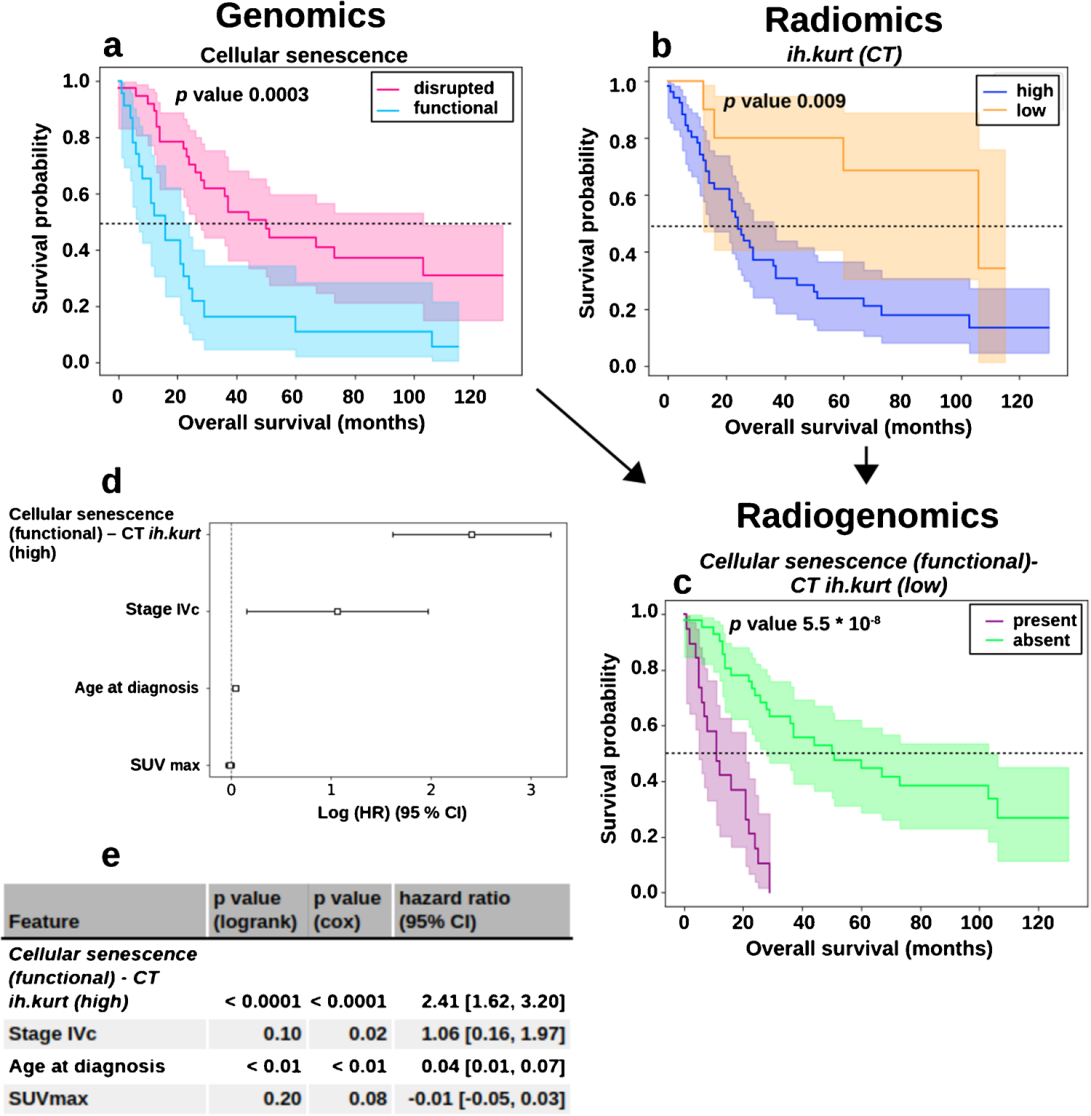
Kaplan–Meier curves for the most prognostic radiogenomic marker and the corresponding univariate markers. Kaplan–Meier curves associated with univariate markers include cellular senescence (**a**) and the CT-based radiomic feature *ih.kurt* (**b**). The Kaplan–Meier curve of the combined radiogenomic marker clearly indicates an improved prognostic stratification (**c**). The radiogenomic marker was defined to be “present” if cellular senescence was functional and the ih.kurt (CT) was low. In all other cases, the radiogenomic marker is “absent.” The forest plot shows the hazard ratios derived using Cox regression (**d**). A summary of the univariate analysis (logrank) and multivariate (cox) analysis with a set of covariates are shown in (**e**)

Multiple cox regression with *cellular senescence (functional)-CT ih.kurt (high)* indicated a strong prognostic value of the radiogenomic marker (*p* < 0.0001, HR 2.41) (Fig. 5d, e). Covariates included age at diagnosis (*p* < 0.01, HR 0.04), SUVmax (*p* 0.08, HR − 0.01), and stage IVc (*p* 0.02, HR 1.06). None of the demographic factors age and gender as well as stage IVc were significantly prognostic in the independent univariate analysis (Supplementary Figs. 10–12).

### Machine learning classification

**Fig. 6:**
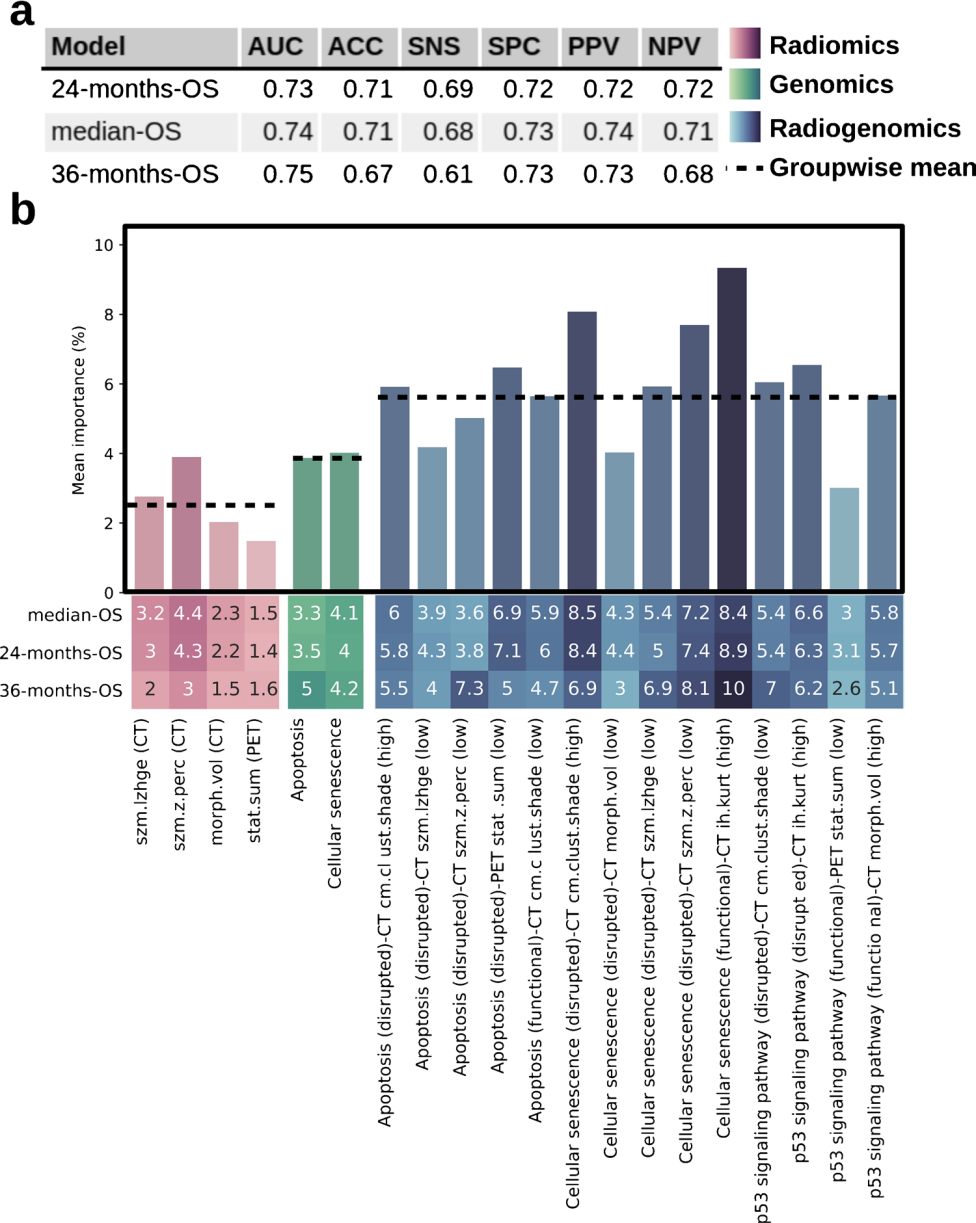
Machine learning–derived feature ranking (% importance) for the three classification models. **a** Performance metrics for the classification models include area under the receiver operating characteristic curve (AUC), accuracy (ACC), sensitivity (SNS), specificity (SPC), positive predictive value (PPV), and negative predictive value (NPV). **b** The heatmap shows the feature contribution for each model. The bar chart shows the importance of each feature as mean over the models. Darker colors indicate higher values. The dashed lines indicate the mean importance over all features belonging to one of the three feature categories, radiomics, genomics, and radiogenomics. Feature importance was calculated based on R-squared ranking

To assess the performance of models integrating complex interactions between multiple genomic, radiomic, and radiogenomic features, a ML approach was employed to establish and cross-validate three binary classifications. Prediction targets were OS greater than 24 months, OS greater than the median OS, and OS greater than 36 months. The cross-validation revealed an area under the receiver operating characteristic curve (AUC) of 0.72 for both the 24-months-OS and the median-OS model. For the 36-months-OS model, a cross-validated AUC of 0.75 was observed. Additional performance metrics are shown in Fig. 6a. Feature importance ranking further indicated the clinical relevance of radiogenomic features, which were the most important attributes in all three models, outperforming genetic as well as radiomic features (Fig. 6b). Over all models, radiomic features had the lowest prognostic value with an average importance of 2.5%, genomic features were associated with an average importance of 4.0%, and radiogenomic features were most important (5.5%).

## Discussion

In our study, we analyzed the association of radiomic with genomic data in HNSCC patients. Our results show a strong influence of the genetic status on quantitative imaging markers in a cohort of HNSCC patients following radiomic and genomic data analysis. By using complementary information from imaging and genetic patterns, we were able to demonstrate that combining radiomic and pathway-level genomic features to radiogenomic markers improves prognostic performance significantly. Furthermore, we identified cellular senescence-derived radiogenomic markers essential for prognostic stratification of HNSCC patients.

In the association analysis of radiomic and genetic traits at the pathway level, we found that higher levels of the PET-based histogram feature *ih.kurt* can be associated with an impaired state of p53 signaling and nitrogen metabolism (Fig. 4). One plausible explanation for the observed association of p53 signaling is the heterogeneous uptake of [^18^F]FDG indicated by *ih.kurt*. The genetic and phenotypic heterogeneity of clonal populations in tumors are the result of an increased number of proliferation cycles, which results in increased mutation rates given the fast growth of tumor tissue [32]. This genetic heterogeneity in clonal populations could be caused by impaired p53 signaling causing genome instability [33]. Genome instability has previously been shown to promote intratumoral heterogeneity detectable on PET via epigenetic mechanisms [34]. Targeting p53 signaling has been shown to be a successful treatment strategy and is currently evaluated in clinical trials using multiple strategies for treating various cancers, including HNSCC [35]. In the association of nitrogen metabolism and the increased metabolism indicated by PET imaging, the amino acid glutamine might play a crucial role. Many cancer cells are reliant on glutamine as main anaplerotic metabolite to fuel the citric acid cycle through a series of biochemical reactions termed glutaminolysis [36]. Therefore, nitrogen metabolism plays an essential role in cells proliferation via anabolic processes such as the biosynthesis of amino acids, nucleotides, and polyamines. Similar to p53 signaling, targeting nitrogen metabolism in proliferating cancer cells has been suggested to be a promising therapeutic approach in clinical studies [37–39]. Considering these aspects, exploring *ih.kurt* as a novel imaging-based marker to determine patients benefitting from these therapeutic approaches is highly promising.

Currently, SUV-based metrics dominate clinical image analysis, given their ease of use and compatibility with conventional PET/CT acquisition protocols. SUV-based metrics have shown prognostic value in a meta-analysis [40]. However, we were not able to reproduce this finding in this study’s cohort. Still, our results identify PET- and CT-derived radiomic features that have prognostic value (Fig. 2a), even where SUV-based metrics did not provide prognostic information in this study’s cohort. Moreover, we identify specific tumor characteristics, which reflect these radiomic features. PET-derived *stat.sum* captures the information of MTV (Fig. 2b, c). This can be explained by PET-derived *stat.sum* indicating the summed activity throughout the entire lesion and consequently is subject to a strong volume-confounding effect [41]. Since *stat.sum* is related to MTV and therefore to the T stage of the tumor, a relation with prognosis is not surprising and presents an expected finding. However, *stat.sum* was slightly more prognostic than MTV, indicating additional prognostic information being captured by the radiomic feature compared to volume alone (Fig. 2d, e). Overall, despite the association of volume-related radiomic features such as *stat.sum* and *morph.vol* with T stage, the investigation of these features is potentially valuable. On the one hand, some of these features provide a fine-grained resolution of the tumor volume itself due to their continuous nature. This makes volume-related radiomic features not only better parameters for automated analysis but also allows for finding optimal thresholds to stratify patients. On the other hand, some volume-related radiomic features such as *stat.sum* incorporate additional information to tumor volume and therefore provide a different viewpoint of the tumor.

The genetically functional state of cellular senescence was significantly associated with reduced survival rates and comprised the most prognostic markers when combined with radiomic features, in the statistical and ML analysis (Figs. 5 and 6). Senescence is known to induce a stable cell cycle arrest triggered by p53 and was therefore proposed as a prevention mechanism for tumorigenesis [42]. However, recent studies have shown that senescent cells can function as tumor promoters, partly due to the proinflammatory and growth-stimulating effects of the senescence-associated secretory phenotype [43].

Since none of the extracted imaging features had a strong association with senescence (Fig. 4), we hypothesized that the identified prognostic imaging markers contain complementary information relevant for prognosis. The ML analysis confirmed the added value of combined radiogenomic features over their univariate counterparts (Fig. 6). Furthermore, the ML analysis demonstrated the capabilities of highly multivariate prediction models as prognostic biomarker (Fig. 5).

Our findings encourage the utilization of senescence-derived radiogenomic markers for the prognostic stratification of HNSCC patients into clinically meaningful groups. Prognosis is certainly one of the most important, yet most difficult issues to address in clinical oncology, not only for the patients but also for their relatives. Prognostic markers, like the ones presented in the present study, can play a vital role in clinical decision-making. They allow for an accurate estimation of prognosis, enabling physicians to anticipate disease progression and, thus, aiding the selection of the most suitable treatment and follow-up scheme and allowing for an optimized allocation of healthcare resources. In addition, the prognostic markers identified in this study provide a primer for research into the mechanistic causes of the survival differences depending on the state of radiogenomic markers.

In our study, mutational tumor DNA was used, delivering a stable and easily reproducible ground truth compared to transcriptomics data deployed in similar radiogenomic studies [44, 45]. In addition, pathway-level genetic markers were used not only integrating information about multiple genes but also deriving information closer to the functional state of the cell. Furthermore, most studies used CT imaging alone [44, 45] while in this study, anatomical information from CT and metabolic information from [^18^F]FDG PET were integrated.

Since we used genetic data derived from solid biopsies, subclonal populations of the tumor cells may not be adequately reflected. To overcome this issue and avoid the drawbacks of surgical interventions, future radiogenomic studies may therefore focus on the use of cell-free DNA (cfDNA) from liquid biopsies to obtain genetic data. Follow-up studies involving DNA sequencing could be greatly simplified, accelerated, and cheapened since panel sequencing focusing on senescence and nitrogen metabolism signaling pathways would be sufficient.

Our study is based on a limited cohort size, which restricted the ML approach to features selected based on the prognostic value in the overall cohort. Since the semi-automated segmentation procedure led to only one segmentation, we were not able to assess the segmentation’s reliability. Furthermore, we were not able to validate our findings using public data since we did not find [^18^F]FDG PET/CT and matched WES data available online. The cohort used in this study is highly heterogeneous, including different clinical subtypes and tumors from multiple locations and stages. Together, this presents a limitation for the translation to clinics since not all findings might be true for all subgroups. The cohort is derived from a single center, requiring an independent, multi-centric validation to account for center-specific biases introduced, for example, through different imaging protocols. Next to imaging protocols, radiomic features are generally sensitive to variations in segmentation protocols and scanner types, creating a challenge when applying radiomic features to other centers.

## Conclusions

In this work, we compared and correlated radiomic with genomic data from HNSCC patients using classical statistics as well as machine learning and were able to find a significant impact of genomic alterations on the corresponding radiomic imaging markers. We demonstrate that combining and unifying PET/CT radiomic and pathway-level genomic features into radiogenomic markers radically improves prognostic performance. In addition, our experiments have revealed the essential role of cellular senescence and derived radiogenomic markers in patient outcome, which may be essential for prognostic stratification of HNSCC patients in the future. Future studies can potentially validate our approach by induction of the presented genetic patterns in preclinical models to investigate the resulting imaging patterns found to be associated with genetic patterns. More research is needed focusing on the investigation of additional data types such as proteomic, epigenomic, and microscopy data to further add to a holistic, personalized picture of cancer patients and improve prognostic biomarkers.

## Supplementary Information

Below is the link to the electronic supplementary material.

Supplementary file1 (DOCX 752 KB)

## Data Availability

The pathway-level genetic, radiomic, standard uptake value (SUV) features and clinical annotation data is publicly available at https://osf.io/rbuqa/.

## Abbreviations

HNSCC: Head and neck squamous cell carcinoma
OS: Overall survival
WES: Whole-exome sequencing
CADD: Combined annotation dependent depletion
VOI: Volume of interest
ML: Machine learning
MTV: Metabolic tumor volume
SUV: Standard uptake value
AUC: Area under the receiver operating characteristic curve
