# Supplementary material for: Radiogenomic markers enable risk stratification and inference of mutational pathway states in head and neck cancer
Source: Eur J Nucl Med Mol Imaging. 2022 Sep 26;50(2):546–58. doi: 10.1007/s00259-022-05973-9 (PMC9816299; doi:10.1007/s00259-022-05973-9)
Supplement: Supplementary file 1 — Supplementary file1 (DOCX 752 KB) [file 259_2022_5973_MOESM1_ESM.docx]

**Supplementary Information**

**Radiogenomic markers enable risk stratification and inference of mutational pathway states in head and neck cancer**

Clemens P. Spielvogel^1,2^, Stefan Stoiber^1,4^, Laszlo Papp^3^, Denis Krajnc^3^, Marko Grahovac^2^, Elisabeth Gurnhofer^4^, Karolina Trachtova^1,2,6^, Vojtech Bystry^6^, Asha Leisser^2^, Bernhard Jank^5^, Julia Schnoell^5^, Lorenz Kadletz^5^, Gregor Heiduschka^5^, Thomas Beyer^3^, Marcus Hacker^2^, Lukas Kenner^1,4^***** & Alexander R. Haug^1,2^*****

*****Corresponding authors

Submitting author： Lukas Kenner (lukas.kenner@meduniwien.ac.at)

^1^Christian Doppler Laboratory for Applied Metabolomics, Vienna, Austria

^2^Division of Nuclear Medicine, Department of Biomedical Imaging and Image-guided Therapy, Medical University of Vienna, Austria

^3^Center for Medical Physics and Biomedical Engineering, Medical University of Vienna, Austria

^4^Clinical Institute of Pathology, Medical University of Vienna, Austria

^5^Department of Otorhinolaryngology, Head and Neck Surgery, Medical University of Vienna, Austria

^6^Centre for Molecular Medicine, [Central European Institute of Technology](https://www.researchgate.net/institution/Central-European-Institute-of-Technology-Czech-Republic), Brno, Czech Repulic

**Supplement section 1**

**DNA extraction and whole exome sequencing**

Histological samples of the primary tumors were retrieved in the form of formalin-fixed paraffin-embedded (FFPE) tissue samples from the tissue archive of the Medical University of Vienna. DNA was isolated from the annotated tumor regions using the EZ1 DNA tissue isolation kit from Qiagen (Cat No./ID: 953034). For 15 samples, tissue annotated as non-tumor tissue was extracted for normalization. The whole exome sequencing (WES) analysis was carried out using sequencing by synthesis on a NovaSeq 6000 sequencer.

**DNA sequencing analysis**

Raw reads were mapped to the genomic reference GRCh38 using the Burrows-Wheeler Alignment (BWA) tool^28^. Small variants were detected using Strelka2^29^ and VarDict^30^ variant callers independently, and the resulting variants were merged. Variants were annotated with the Variant Effect Predictor (VEP) tool from Ensemble^31^ including the annotation of CADD scores^32–34^. Resulting annotated variants were joined across the cohort and germline variants were filtered. The discrimination of somatic and germline variants was based on a somatic tumor variant filtering strategy from Sukhai et al.^35^, with additional filters added and parameters adjusted in order to minimize the ratio of known germline variants resulting from a set of 15 paired normal tissues. The final somatic variant filtering was performed as follows. Only variants present in less than 10% of samples were kept. Variants called by both Strelka2 and VarDict with a number of variant reads above 10 and variants called by only one variant caller with a number of variant reads above 20 were kept. Three population variant databases were used for variant filtering including 1000 genome^36^, Gnomad^37^, and the NHLBI Exome Sequencing Project^38^. Variants with a minor allele frequency below 1% for the non-finnish European group in all three databases were kept. Variants with a record in ClinVar database^39^ with significance "benign" or "likely benign" were removed.

**Radiomic feature extraction and preprocessing**

The standard uptake values (SUVs) for each delineated lesion voxel were normalized using the SUVmean of the respective reference background region in PET images. The anatomical reference background region was the aortic arch. Resampling was carried out using Kriging interpolation at two millimeters and four millimeters^42^. The tumor characteristics quantified by the resulting features corresponded to four different types including intensity, histogram, shape and texture. Textural features were derived based on three matrix types, namely grey-level co-occurrence matrix (GLCM), grey-level zone size matrix (GLSZM) and neighborhood grey-tone difference matrix (NGTDM). For CT, features were derived in a similar way to PET-based features, although no background normalization was applied. Only robust features were further processed^43^. Only features having ‘strong’ and ‘very strong’ multi-centric consensus as defined by IBSI were selected for analysis to support repeatability of the findings. To ensure comparability between radiomic features and genetic features, radiomic features were only extracted from the suspected primary lesions. Using this procedure, a total of 104 radiomic features were extracted, 52 from PET and CT respectively. All radiomic features are denoted by their IBSI unique ID (Supplementary Table 4). Image acquisition, delineation and feature extraction has been performed in accordance with the IBSI reporting standard (Supplementary table 5)^44^. In order to only assess high quality radiomic features, no radiomic features were computed if lesions had a size below 64 voxels^45^. Further, a redundancy removal step, where pairwise correlations between all features were assessed and for feature pairs with a correlation coefficient above 0.66, the feature with the higher variance from the pairs was selected for further analysis.

In addition to radiomic features, we extracted SUV metrics from the PET images using the aforementioned Hybrid 3D software. Two patients with missing SUV metrics were excluded from the respective analysis. The SUV-based metrics included SUVmax, SUVmin, SUVmean, SUVpeak and SUV TLG which were all normalized to body weight.

**Code and visualization tools**

All analyses were conducted using Python 3. Packages used included pandas 1.0.3, numpy 1.19.2 and scikit-learn 0.23.2. For the survival analysis and plotting of associated Kaplan-Meier curves, lifelines 0.24.13 was used. For any other statistical analysis, we used SciPy 1.4.1. Visualizations were created using Matplotlib 3.2.1 and Seaborn 0.11.1. For the creation of rain cloud plots, we used the package Ptprince 0.2. For the creation of sankey diagrams, Plotly 4.4.1 was used. The graphical abstract was created using BioRender (biorender.com).


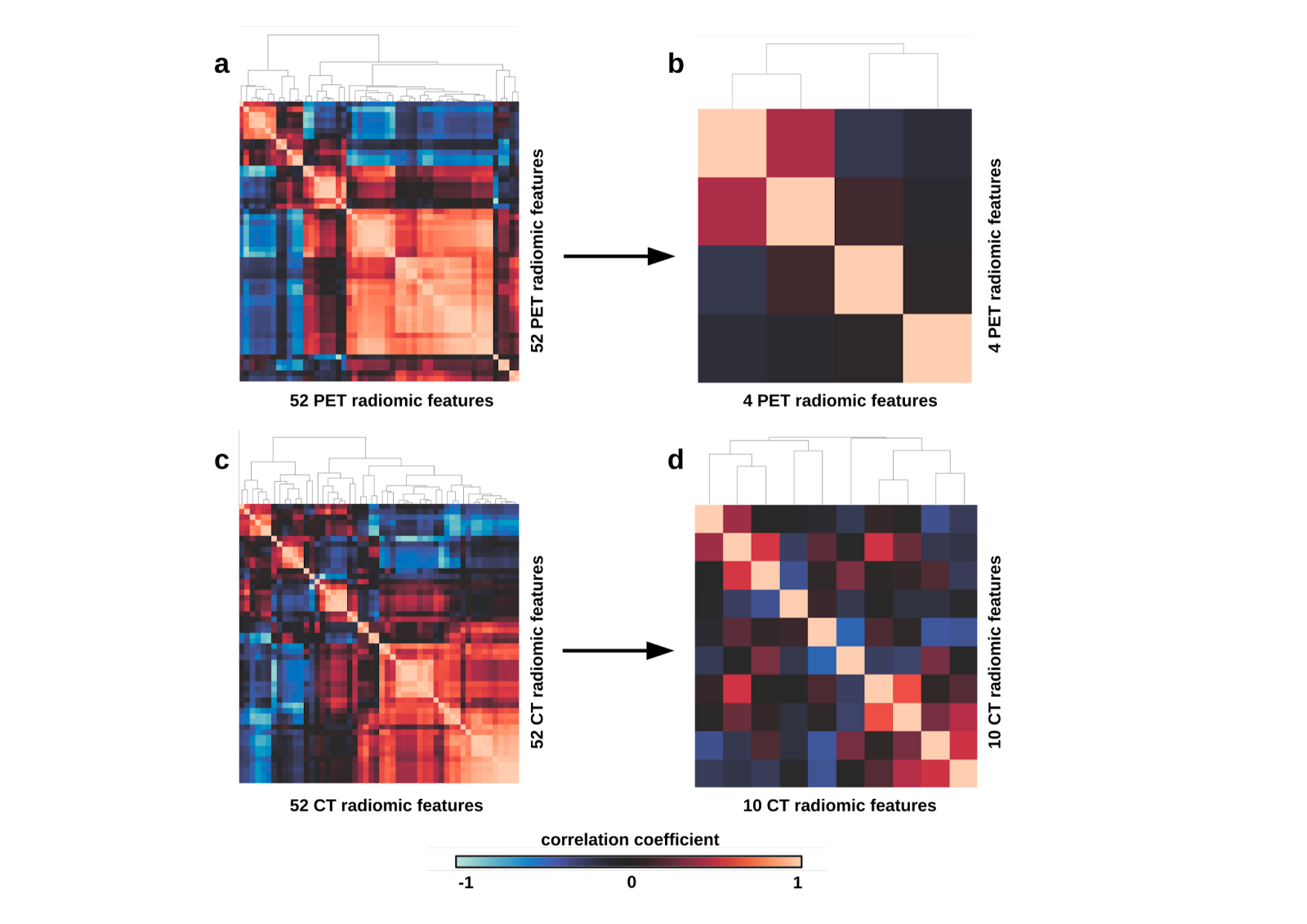


**Supplementary Fig. 1:** **Correlation-based feature selection for PET or CT-derived radiomic features.** Reduced CT features contained two histogram, one intensity, one morphological and six texture features. PET features included one intensity, one histogram and two textural features. The feature with the lower variance was kept for further analysis if there was a feature pair with a correlation coefficient above 0.66.


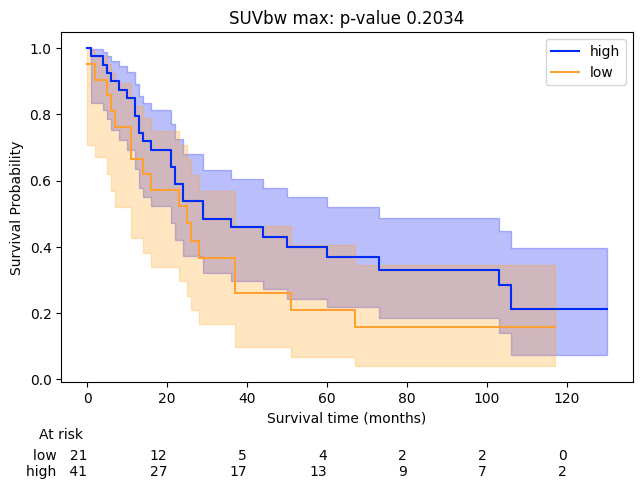
 **Supplementary Fig. 2: Kaplan-Meier curve for SUVmax (*p* 0.20).
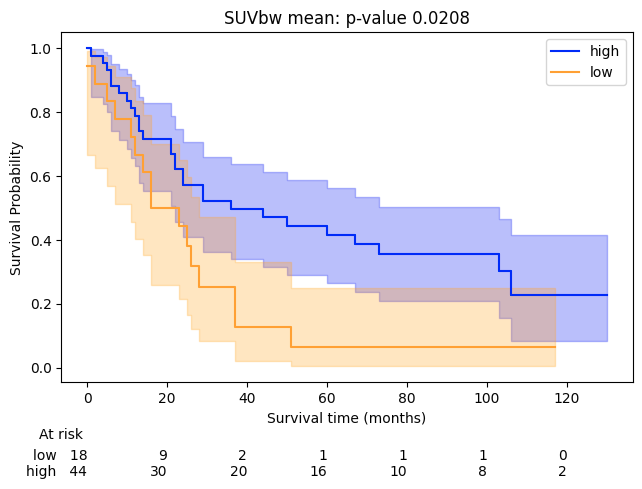
 Supplementary Fig. 3: Kaplan-Meier curve for SUVmean (*p* 0.02).**

**
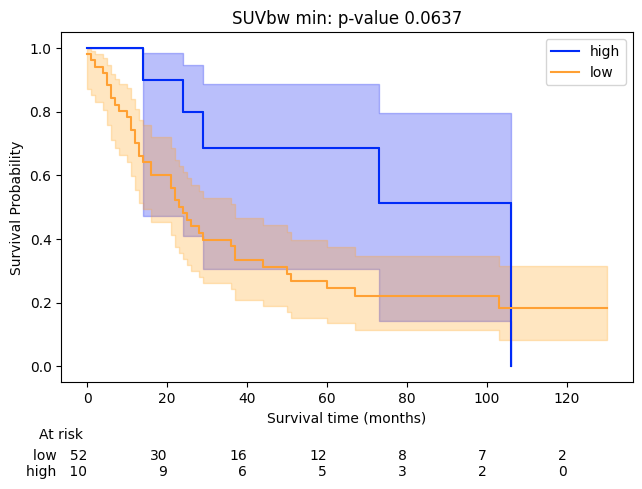
 Supplementary Fig. 4: Kaplan-Meier curve for SUVmin (*p* 0.06).
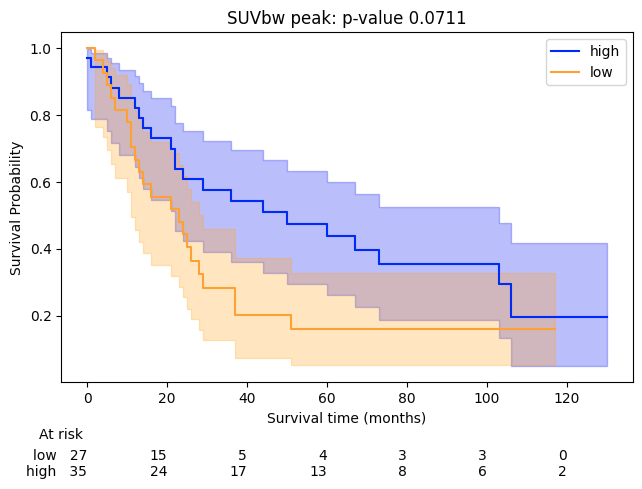
 Supplementary Fig. 5: Kaplan-Meier curve for SUVpeak (p 0.07).**

**
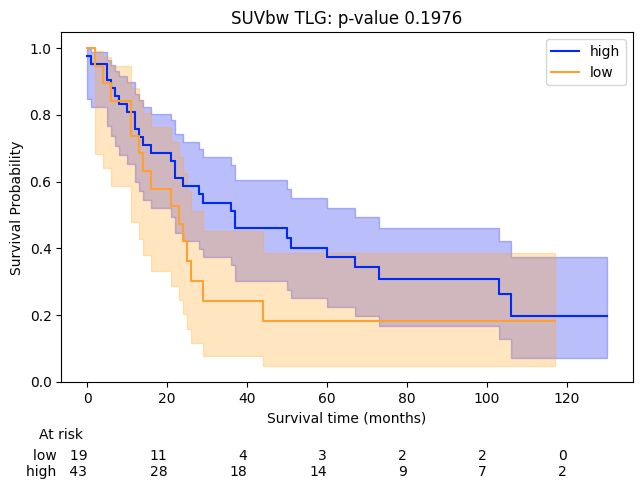
 Supplementary Fig. 6: Kaplan-Meier curve for SUV TLG (*p* 0.20).**


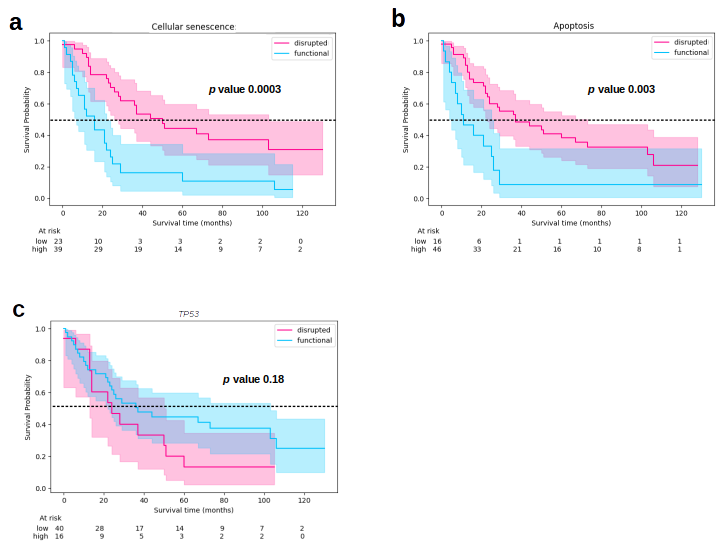


**Supplementary Fig. 7: Kaplan-Meier curves for the two pathways related to cellular growth and death. (a, b) significant after Bonferroni correction as well as for the *TP53* gene (c).** a, cellular senescence. b, apoptosis. c, *TP53*. Even though *TP53* is a strong influencing factor for the pathway-level CADD scores of both prognostic pathways, the pathway-level scores provide a superior prognostic value.


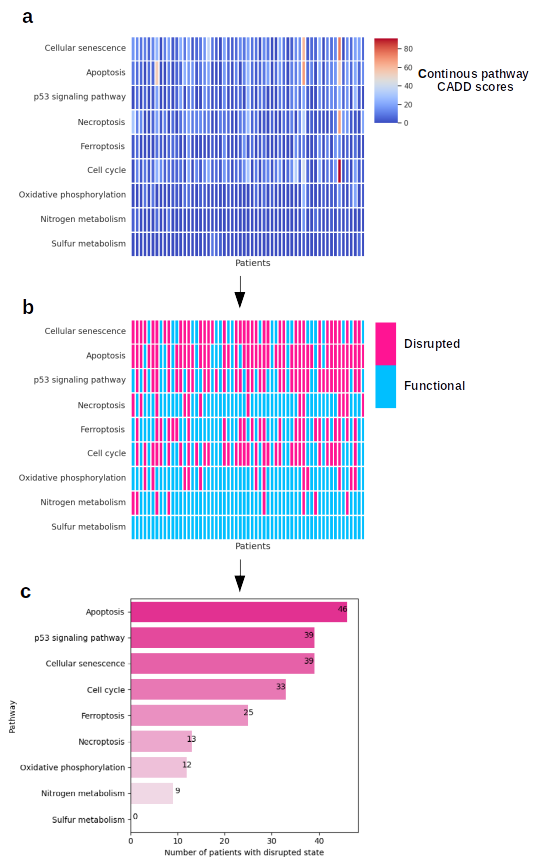


**Supplementary Fig. 8: Binarization of the continuous pathway disruption scores.** a, continuous pathway CADD scores. b, binarized pathway CADD scores. c, Number of disrupted pathways among patients. First, continuous pathway scores were derived. Second, a survival analysis with an optimized cutoff was conducted. Third, the pathways were associated into one of two groups, functional or disrupted, based on the most prognostically relevant cutoff determined by survival analysis.


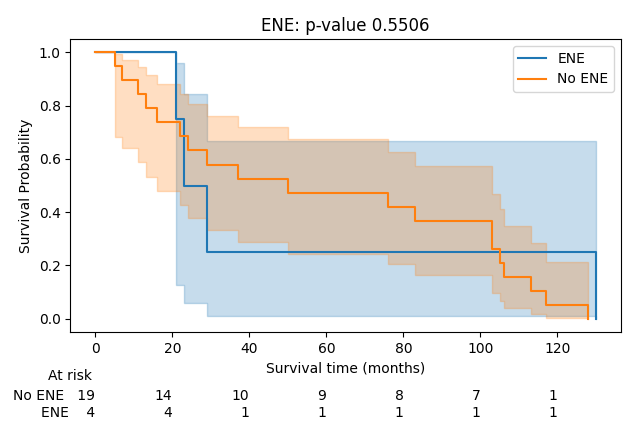
 **Supplementary Fig. 9: Kaplan-Meier curve for extranodal extension (ENE) (*p* 0.55).**


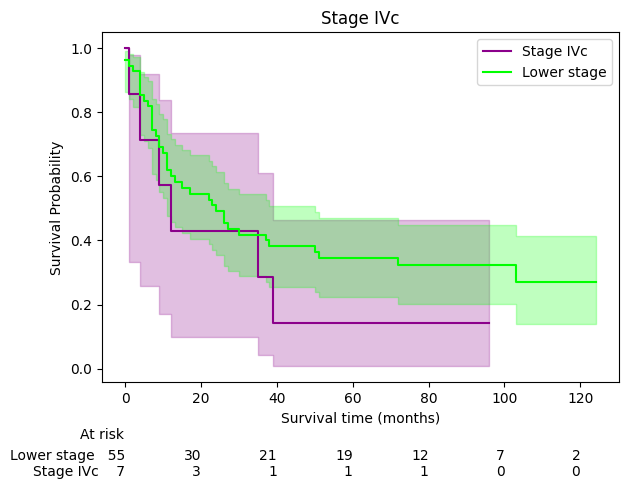


**Supplementary Fig. 10: Kaplan-Meier curve for stage IVc (*p* 0.35).**


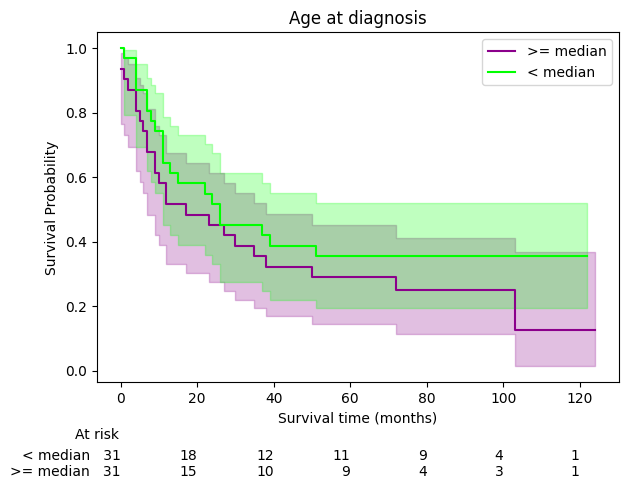


**Supplementary Fig. 11: Kaplan-Meier curve for age at diagnosis (*p* 0.26).**


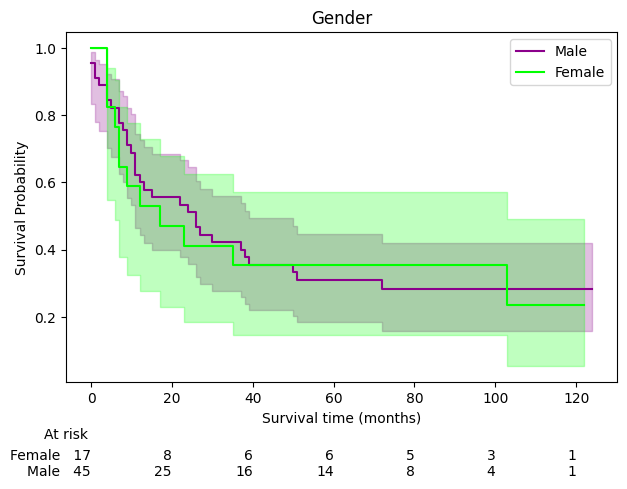


**Supplementary Fig. 12: Kaplan-Meier curve for gender (*p* 0.95).**

*
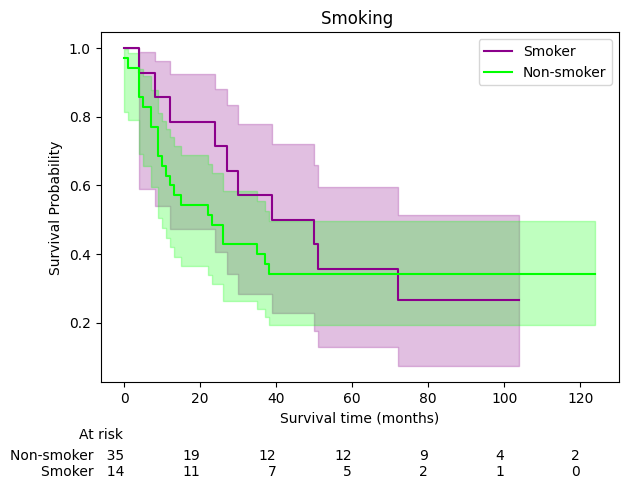
*

**Supplementary Fig. 13: Kaplan-Meier curve smoking (*p* 0.66).**

| **Supplementary Table 1: Survival analysis results of commonly used SUV metrics.Feature** | ***p* value** |
| --- | --- |
| SUV mean | 0.02 |
| SUV min | 0.06 |
| SUV peak | 0.07 |
| SUV TLG | 0.20 |
| SUV max | 0.20 |

| **Supplementary Table 2. List of associations analysis results for radiomic features and pathways.Pathway disruption** | **Radiomic feature** | ***p* value** |
| --- | --- | --- |
| p53 signaling pathway | PET::Histogram::ih.kurt | 0.001 |
| Nitrogen metabolism | PET::Histogram::ih.kurt | 0.003 |
| Nitrogen metabolism | PET::GLCM::cm.clust.prom | 0.027 |
| Ferroptosis | CT::Intensity::stat.sum | 0.031 |
| Ferroptosis | PET::Histogram::ih.kurt | 0.114 |
| p53 signaling pathway | CT::GLSZM::szm.lzhge | 0.119 |
| Ferroptosis | CT::GLCM::cm.clust.shade | 0.132 |
| p53 signaling pathway | PET::GLCM::cm.clust.prom | 0.145 |
| Nitrogen metabolism | CT::Histogram::ih.kurt | 0.174 |
| Cellular senescence | PET::GLCM::cm.clust.prom | 0.210 |
| p53 signaling pathway | CT::GLSZM::szm.lzlge | 0.210 |
| Cellular senescence | PET::Intensity::stat.sum | 0.226 |
| Cellular senescence | CT::Histogram::ih.skew | 0.226 |
| Cellular senescence | CT::Morphological::morph.vol | 0.253 |
| Apoptosis | PET::Histogram::ih.kurt | 0.257 |
| Nitrogen metabolism | CT::GLSZM::szm.lzlge | 0.318 |
| Cellular senescence | CT::GLSZM::szm.lzlge | 0.322 |
| Nitrogen metabolism | CT::GLCM::cm.clust.shade | 0.327 |
| p53 signaling pathway | CT::Histogram::ih.skew | 0.329 |
| Cellular senescence | CT::Histogram::ih.kurt | 0.351 |
| Apoptosis | CT::GLCM::cm.clust.prom | 0.372 |
| Apoptosis | CT::NGTDM::ntg.complexity | 0.372 |
| p53 signaling pathway | PET::GLSZM::szm.lze | 0.378 |
| Ferroptosis | CT::GLSZM::szm.lzlge | 0.422 |
| Ferroptosis | CT::Histogram::ih.kurt | 0.438 |
| Nitrogen metabolism | CT::Intensity::stat.sum | 0.460 |
| p53 signaling pathway | CT::GLCM::cm.clust.shade | 0.466 |
| Cellular senescence | CT::GLCM::cm.clust.prom | 0.466 |
| Nitrogen metabolism | CT::Histogram::ih.skew | 0.472 |
| Cellular senescence | CT::NGTDM::ntg.complexity | 0.493 |
| Apoptosis | PET::GLSZM::szm.lze | 0.494 |
| Ferroptosis | PET::GLSZM::szm.lze | 0.556 |
| Ferroptosis | PET::GLCM::cm.clust.prom | 0.556 |
| Ferroptosis | CT::GLSZM::szm.lzhge | 0.595 |
| Ferroptosis | CT::Morphological::morph.vol | 0.600 |
| Apoptosis | CT::GLSZM::szm.lzlge | 0.647 |
| Cellular senescence | PET::Histogram::ih.kurt | 0.694 |
| p53 signaling pathway | PET::Intensity::stat.sum | 0.694 |
| p53 signaling pathway | CT::NGTDM::ntg.complexity | 0.694 |
| Apoptosis | CT::GLCM::cm.clust.shade | 0.729 |
| Apoptosis | PET::Intensity::stat.sum | 0.766 |
| Apoptosis | CT::Intensity::stat.sum | 0.766 |
| Apoptosis | CT::Histogram::ih.kurt | 0.791 |
| Ferroptosis | PET::Intensity::stat.sum | 0.796 |
| Ferroptosis | CT::Histogram::ih.skew | 0.796 |
| Nitrogen metabolism | PET::GLSZM::szm.lze | 0.810 |
| Nitrogen metabolism | PET::GLSZM::szm.lze | 0.810 |
| Apoptosis | CT::GLSZM::szm.lzhge | 0.816 |
| Cellular senescence | PET::GLSZM::szm.lze | 0.816 |
| p53 signaling pathway | CT::GLCM::cm.clust.prom | 0.816 |
| Nitrogen metabolism | PET::Intensity::stat.sum | 0.826 |
| Nitrogen metabolism | PET::Intensity::stat.sum | 0.826 |
| p53 signaling pathway | CT::Histogram::ih.kurt | 0.827 |
| p53 signaling pathway | CT::Morphological::morph.vol | 0.855 |
| Nitrogen metabolism | CT::GLCM::cm.clust.prom | 0.857 |
| Apoptosis | CT::Histogram::ih.skew | 0.866 |
| Cellular senescence | CT::Intensity::stat.sum | 0.873 |
| Cellular senescence | CT::GLSZM::szm.lzhge | 0.873 |
| Nitrogen metabolism | CT::NGTDM::ntg.complexity | 0.873 |
| p53 signaling pathway | CT::Intensity::stat.sum | 0.896 |
| Apoptosis | PET::GLCM::cm.clust.prom | 0.904 |
| Nitrogen metabolism | CT::Morphological::morph.vol | 0.905 |
| Ferroptosis | CT::NGTDM::ntg.complexity | 0.954 |
| Ferroptosis | CT::GLCM::cm.clust.prom | 0.966 |
| Apoptosis | CT::Morphological::morph.vol | 0.974 |
| Nitrogen metabolism | CT::GLSZM::szm.lzhge | 0.984 |
| Cellular senescence | CT::GLCM::cm.clust.shade | 0.988 |

| **Supplementary Table 3: Prognostic value of radiogenomic features.** Radiogenomic feature postfixes contain two numbers. The first one indicates the functional state of the pathway, the second indicates whether the radiomic feature is low or high. 0 indicates functional and low respectively. 1 indicates disrupted and high respectively. Feature pairs are ranked by their *p value* (logrank). **Feature** | ***p-value* (logrank)** | ***p-value* (cox regression)** | **hazard ratio (HR)** | **HR 95% CI lower** | **HR 95% CI upper** | **median survival (high)** | **median survival (low)** |
| --- | --- | --- | --- | --- | --- | --- | --- |
| Cellular senescence-CT::Histogram::ih.kurt-0-1 | 5.47E-08 | 1.04772E-06 | 6.13 | 1.09 | 2.54 | 11 | 51 |
| Cellular senescence-CT::GLSZM::szm.z.perc-0-0 | 0.000000469 | 6.95888E-06 | 6.01 | 1.01 | 2.57 | 8 | 37 |
| Cellular senescence-CT::GLSZM::szm.lzhge-1-0 | 0.0000103 | 3.25356E-05 | 0.26 | -1.97 | -0.71 | 67 | 14 |
| Apoptosis-CT::GLSZM::szm.lzhge-1-0 | 0.0000478 | 0.000107693 | 0.3 | -1.83 | -0.6 | 51 | 12 |
| Cellular senescence-PET::Intensity::stat.sum-1-0 | 0.000052 | 0.000127544 | 0.28 | -1.91 | -0.62 | 73 | 14 |
| Apoptosis-CT::GLSZM::szm.z.perc-1-1 | 0.0000711 | 0.000165504 | 0.29 | -1.87 | -0.59 | 67 | 16 |
| Cellular senescence-CT::GLCM::cm.clust.shade-1-1 | 0.0000921 | 0.000190786 | 0.31 | -1.8 | -0.56 | 51 | 13 |
| Cellular senescence-CT::Morphological::morph.vol-1-0 | 0.000135263 | 0.000266634 | 0.31 | -1.79 | -0.54 | 67 | 14 |
| Apoptosis-PET::Intensity::stat.sum-1-0 | 0.000219464 | 0.00039472 | 0.33 | -1.7 | -0.49 | 51 | 12 |
| Cellular senescence-CT::GLSZM::szm.z.perc-1-1 | 0.000316254 | 0.000681342 | 0.29 | -1.95 | -0.52 | 103 | 21 |
| p53 signaling pathway-CT::Histogram::ih.kurt-0-1 | 0.000398597 | 0.000755915 | 3.12 | 0.48 | 1.8 | 12 | 44 |
| Apoptosis-CT::Morphological::morph.vol-1-0 | 0.000464155 | 0.000748803 | 0.35 | -1.64 | -0.44 | 51 | 12 |
| p53 signaling pathway-CT::GLSZM::szm.lzhge-1-0 | 0.000551227 | 0.000897221 | 0.36 | -1.63 | -0.42 | 51 | 14 |
| p53 signaling pathway-CT::GLSZM::szm.z.perc-0-0 | 0.000641859 | 0.001163434 | 3.07 | 0.44 | 1.8 | 12 | 37 |
| Apoptosis-CT::GLCM::cm.clust.shade-1-1 | 0.00113921 | 0.001713399 | 0.38 | -1.56 | -0.36 | 44 | 13 |
| Cellular senescence-CT::Histogram::ih.skew-1-1 | 0.001469007 | 0.002041461 | 0.38 | -1.6 | -0.36 | 51 | 21 |
| Apoptosis-CT::Histogram::ih.skew-1-1 | 0.004001362 | 0.005201185 | 0.41 | -1.51 | -0.27 | 50 | 21 |
| Apoptosis-CT::Histogram::ih.kurt-0-1 | 0.005591924 | 0.007600188 | 2.52 | 0.25 | 1.61 | 11 | 37 |
| Cellular senescence-CT::GLCM::cm.clust.shade-0-1 | 0.007367971 | 0.008623882 | 2.33 | 0.21 | 1.48 | 16 | 37 |
| p53 signaling pathway-CT::GLSZM::szm.z.perc-1-1 | 0.007967131 | 0.009962344 | 0.44 | -1.46 | -0.2 | 50 | 21 |
| p53 signaling pathway-PET::Intensity::stat.sum-1-0 | 0.009213384 | 0.011123015 | 0.46 | -1.39 | -0.18 | 51 | 16 |
| p53 signaling pathway-CT::Morphological::morph.vol-1-0 | 0.018019028 | 0.020538042 | 0.49 | -1.31 | -0.11 | 50 | 16 |
| p53 signaling pathway-CT::GLCM::cm.clust.shade-1-1 | 0.020120649 | 0.023028641 | 0.5 | -1.29 | -0.1 | 44 | 16 |
| p53 signaling pathway-CT::Histogram::ih.skew-1-1 | 0.020570542 | 0.023023638 | 0.49 | -1.31 | -0.1 | 51 | 22 |
| Apoptosis-CT::GLSZM::szm.z.perc-0-0 | 0.035228544 | 0.042470658 | 2.23 | 0.03 | 1.58 | 10 | 36 |
| Apoptosis-CT::GLSZM::szm.z.perc-1-0 | 0.040464555 | 0.043036774 | 1.91 | 0.02 | 1.28 | 21 | 29 |
| Apoptosis-CT::GLCM::cm.clust.shade-0-1 | 0.043505157 | 0.048663769 | 2.06 | 0 | 1.44 | 11 | 36 |
| Cellular senescence-CT::GLSZM::szm.lzhge-0-0 | 0.053291834 | 0.055526052 | 1.85 | -0.01 | 1.25 | 21 | 37 |
| Cellular senescence-PET::Intensity::stat.sum-0-0 | 0.066861539 | 0.068900333 | 1.8 | -0.05 | 1.22 | 21 | 37 |
| Cellular senescence-CT::Morphological::morph.vol-0-0 | 0.066861539 | 0.068900333 | 1.8 | -0.05 | 1.22 | 21 | 37 |
| Cellular senescence-CT::Histogram::ih.skew-0-1 | 0.070115415 | 0.072671022 | 1.8 | -0.05 | 1.23 | 16 | 36 |
| Apoptosis-PET::Intensity::stat.sum-1-1 | 0.074500078 | 0.076730696 | 1.91 | -0.07 | 1.36 | 12 | 29 |
| Apoptosis-CT::Morphological::morph.vol-1-1 | 0.123777104 | 0.124559735 | 1.84 | -0.17 | 1.38 | 12 | 29 |
| Apoptosis-CT::Histogram::ih.skew-0-1 | 0.127621331 | 0.135718487 | 1.81 | -0.19 | 1.37 | 11 | 29 |
| Apoptosis-PET::Intensity::stat.sum-0-0 | 0.154910283 | 0.161214099 | 1.74 | -0.22 | 1.33 | 16 | 29 |
| Apoptosis-CT::Morphological::morph.vol-0-0 | 0.154910283 | 0.161214099 | 1.74 | -0.22 | 1.33 | 16 | 29 |
| Cellular senescence-CT::Histogram::ih.kurt-1-1 | 0.160503337 | 0.161841665 | 0.65 | -1.02 | 0.17 | 37 | 21 |
| Apoptosis-CT::GLSZM::szm.lzhge-0-0 | 0.175516825 | 0.178212112 | 1.7 | -0.24 | 1.31 | 16 | 29 |
| Cellular senescence-PET::Intensity::stat.sum-1-1 | 0.245309665 | 0.245048034 | 1.55 | -0.3 | 1.18 | 14 | 28 |
| p53 signaling pathway-CT::GLCM::cm.clust.shade-0-1 | 0.387280524 | 0.391925376 | 1.33 | -0.37 | 0.93 | 23 | 29 |
| p53 signaling pathway-CT::GLSZM::szm.z.perc-1-0 | 0.390513972 | 0.392761174 | 1.35 | -0.39 | 0.98 | 21 | 29 |
| p53 signaling pathway-CT::Histogram::ih.skew-0-1 | 0.415879788 | 0.419131018 | 1.32 | -0.39 | 0.94 | 23 | 29 |
| p53 signaling pathway-CT::GLSZM::szm.lzhge-0-0 | 0.418120773 | 0.422338634 | 1.34 | -0.42 | 1 | 22 | 29 |
| Cellular senescence-CT::GLSZM::szm.z.perc-0-1 | 0.419805744 | 0.415760935 | 1.34 | -0.42 | 1 | 22 | 28 |
| Cellular senescence-CT::GLSZM::szm.z.perc-1-0 | 0.585554645 | 0.589668536 | 1.2 | -0.47 | 0.83 | 26 | 28 |
| Apoptosis-CT::Histogram::ih.kurt-1-1 | 0.707463285 | 0.700625015 | 1.13 | -0.5 | 0.74 | 28 | 25 |
| p53 signaling pathway-CT::Histogram::ih.kurt-1-1 | 0.786228028 | 0.791467387 | 0.92 | -0.68 | 0.52 | 29 | 23 |
| p53 signaling pathway-CT::Morphological::morph.vol-0-0 | 0.861213642 | 0.868521824 | 1.06 | -0.65 | 0.77 | 25 | 29 |
| p53 signaling pathway-PET::Intensity::stat.sum-0-0 | 0.861213642 | 0.868521824 | 1.06 | -0.65 | 0.77 | 25 | 29 |

| **Supplementary Table 4: Abbreviations and full names for all 52 extracted radiomic features.Abbreviation** | **Full name** |
| --- | --- |
| cm.auto.corr | Autocorrelation |
| cm.clust.prom | Cluster prominence |
| cm.clust.shade | Cluster shade |
| cm.contrast | Contrast |
| cm.corr | Correlation |
| cm.diff.avg | Difference Average |
| cm.diff.entr | Difference entropy |
| cm.diff.var | Difference variance |
| cm.dissimilarity | Dissimilarity |
| cm.energy | Angular second moment |
| cm.info.corr.1 | Information correlation 1 |
| cm.inv.diff | Inverse difference |
| cm.inv.diff.mom | Inverse difference moment |
| cm.joint.avg | Joint average |
| cm.joint.entr | Joint entropy |
| cm.joint.max | Joint maximum |
| cm.joint.var | Joint variance |
| cm.sum.avg_x | Sum average |
| cm.sum.avg_y | Sum average |
| cm.sum.entr | Sum entropy |
| cm.sum.var | Sum variance |
| ih.entropy | Discretised intensity entropy |
| ih.kurt | (Excess) discretised intensity kurtosis |
| ih.mean | Mean discretised intensity |
| ih.skew | Discretised intensity skewness |
| ih.uniformity | Discretised intensity uniformity |
| ih.var | Discretised intensity variance |
| morph.area | Surface area (mesh) |
| morph.comp.1 | Compactness 1 |
| morph.sph.dispr | Spherical disproportion |
| morph.vol | Volume (mesh) |
| ngt.busyness | Busyness |
| ngt.coarseness | Coarseness |
| ngt.contrast | Contrast |
| ngt.strength | Strength |
| ntg.complexity | Complexity |
| stat.max | Maximum intensity |
| stat.mean | Mean intensity |
| stat.min | Minimum intensity |
| stat.range | Intensity range |
| stat.sum | Sum intensity |
| stat.var | Intensity variance |
| szm.glnu | Grey level non-uniformity |
| szm.hgze | High grey level zone emphasis |
| szm.lgze | Low grey level zone emphasis |
| szm.lze | Large zone emphasis |
| szm.lzhge | Large zone high grey level emphasis |
| szm.lzlge | Large zone low grey level emphasis |
| szm.sze | Small zone emphasis |
| szm.szhge | Small zone high grey level emphasis |
| szm.z.perc | Zone percentage |
| szm.zsnu | Zone size non-uniformity |

| **Supplementary Table 5: Image biomarker standardization initiative (IBSI) reporting table.Patient** | |
| --- | --- |
| Volume of Interest | Histologically confirmed 18-F-FDG PET/CT-positive head and neck squamous cell carcinoma lesions |
| Patient Preparation | Patients were required to fast for at least 5 h before injection of (200–350) MBq 18FFDG based on body weight with blood glucose level <150 mg/dL (8.3 mmol/L). |
| Radiotracer | 18F-FDG |
| **Acquisition and Reconstruction** | |
| Protocol | A dedicated breast PET/CT scan was performed over one PET bed position with the patient in the prone position. |
| Scanner type | Biograph 64 PET/CT System (Siemens Healthineers, Erlangen, Germany) |
| [^18^F]FDG | - Static - 3 min bed position - 30 min after application - 4.08 x 4.08 x 5.00 mm voxel size |
| CT | - Static - 1.37 x 1.37 x 5 mm voxel size |
| [^18^F]FDG: 2-Deoxy-2-[^18^F]fluoroglucose, CT: Computed tomography | |
| **Image Co-registration** | |
| Software | Hermes Hybrid 3D ver 4.0 |
| Co-registration step 1 | Automated as of DICOM coordinate parameters |
| **Data conversion** | |
| Step 1 (all images) | Initial voxel values determined as of transforming the DICOM raw voxel values by the DICOM tags Rescale Scope (0028\|1053) and Rescale Intercept (0028\|1052). |
| Step 2  [^18^F]FDG | Initial voxel values transformed to tumor-to-background ratio (TBR) by dividing all voxel values with the mean of the reference region drawn as a 9x9x9 cuboid VOI in the ??? region in each patient. |
| **Segmentation** | |
| Software | Hermes Hybrid 3D ver 4.0 |
| VOI definition | Standard semi-automated iso-count 3D based on side-by-side viewing PET/CT. |
| Number of experts | 2 nuclear medicine experts participated in independent delineations, followed by 1 senior nuclear medicine specialist cross-validation and if necessary, modification of first-round results. |
| Reference image | [^18^F]FDG PET |
| **Image / VOI interpolation** | |
| Method | Kriging interpolation in 3D (2), including nearest neighbors within distance of voxel size main diagonal + epsilon. |
| Grid | Align by center |
| Extrapolation beyond original image | Neighbor distance search calculated as original voxel size main diagonal + epsilon. Missing value: image minimum |
| Partially masked voxels (VOI) | Taken if more than half of original voxel area included |
| **Discretization** | |
| Method | Fixed bin width, variable number of bins |
| Bin width | - [^18^F]FDG: 0.05 (in TBR units) - CT: 2 (in original units) |
| **Image biomarker computation / Parameters** | |
| Biomarker set | The following 52 features were extracted for PET and CT image respectively:  Autocorrelation  Cluster prominence  Cluster shade  Contrast  Correlation  Difference Average  Difference entropy  Difference variance  Dissimilarity  Angular second moment  Information correlation 1  Inverse difference  Inverse difference moment  Joint average  Joint entropy  Joint maximum  Joint variance  Sum average  Sum average  Sum entropy  Sum variance  Discretised intensity entropy  (Excess) discretised intensity kurtosis  Mean discretised intensity  Discretised intensity skewness  Discretised intensity uniformity  Discretised intensity variance  Surface area (mesh)  Compactness 1  Spherical disproportion  Volume (mesh)  Busyness  Coarseness  Contrast  Strength  Complexity  Maximum intensity  Mean intensity  Minimum intensity  Intensity range  Sum intensity  Intensity variance  Grey level non-uniformity  High grey level zone emphasis  Low grey level zone emphasis  Large zone emphasis  Large zone high grey level emphasis  Large zone low grey level emphasis  Small zone emphasis  Small zone high grey level emphasis  Zone percentage  Zone size non-uniformity |
| Software | MUW radiomics engine (3) ver. 2.0, developed and validated based on IBSI guidelines and reference datasets (1).  Software availability upon reasonable request from the corresponding author. |
| Distance weighting | No |
| CM symmetry | Symmetric |
| CM / ZM distance | Chebyshev distance 1 |
| CM / ZM aggregation | 3D, full-merging |
| Exclusion criteria | VOIs with less than 64 voxels were excluded from the analysis |

| **Supplementary Table 6: In- and excluded KEGG pathways.Pathway name** | **Function** | **Excluded** |
| --- | --- | --- |
| Oxidative phosphorylation | Energy metabolism | No |
| Nitrogen metabolism | Energy metabolism | No |
| Sulfur metabolism | Energy metabolism | No |
| Photosynthesis | Energy metabolism | Yes |
| Photosynthesis - antenna proteins | Energy metabolism | Yes |
| Carbon fixation in photosynthetic organisms | Energy metabolism | Yes |
| Carbon fixation pathways in prokaryotes | Energy metabolism | Yes |
| Methane metabolism | Energy metabolism | Yes |
| Cell cycle | Cell growth and death | No |
| Cellular senescence | Cell growth and death | No |
| Apoptosis | Cell growth and death | No |
| Ferroptosis | Cell growth and death | No |
| Necroptosis | Cell growth and death | No |
| p53 signaling pathway | Cell growth and death | No |
| Oocyte meiosis | Cell growth and death | Yes |
| Cell cycle - yeast | Cell growth and death | Yes |
| Cell cycle - Caulobacter | Cell growth and death | Yes |
| Meiosis - yeast | Cell growth and death | Yes |
| Apoptosis - fly | Cell growth and death | Yes |
| Apoptosis - multiple species | Cell growth and death | Yes |

**Supplement section 2**

Machine learning analysis for the 24-months-OS classification model

**AUTOMATED MACHINE LEARNING ANALYSIS**

Tabular data submission for Dedicaid AutoML services was performed by Dedicaid user clemens.spielvogel@meduniwien.ac.at on 8/1/2021, 3:59:19 PM to build and cross-validate automated data preprocessing and mixed, stacked ensemble learning pipelines for predicting reference label OS_histo24. For the details of the analysis see Supplementary Table 7.

**Supplementary Table 7.** Properties of the automated machine learning (AutoML) analysis of this study.

| Data name | fdb_multiomics_w_labels_bonferroni_significant.xlsx |
| --- | --- |
| Data size | 62 samples, 20 features |
| Date of analysis | 8/1/2021, 3:59:19 PM |
| Duration of analysis | 3h 1m 36s |
| Submitted by | clemens.spielvogel@meduniwien.ac.at |
| Dedicaid AutoML version | 0.1 |

**METHODS**

**Data**

The input dataset was composed of 62 samples and 23 features. The submitted dataset was composed of 20 features. The selected reference label for the cross-validation was OS_histo24 having subgroups of 0 (50%) and 1 (50%) label outcomes.

**Cross-Validation**

Monte Carlo (MC) cross-validation scheme was applied with 80% training and 20% validation ratios across 100 folds (3). Each fold had unique training-validation configurations. MC split resulted in 50 samples per fold in the training set. The validation set of each fold contained 6 samples per reference label (12 overall). The validation samples were equally subsampled to ensure that none of the label outcomes are over or underrepresented during the cross-validation.

**Preprocessing**

The data underwent preprocessing steps in each fold before performing machine learning (ML) analysis. Preprocessing resulted in average 84 samples and 13 features across all MC folds. For the preprocessing steps and their parameters, see Supplementary Table 8.

**Supplementary Table 8.** Preprocessing step algorithms as well as their parameter values performed in all Monte Carlo folds before machine learning. FN - Feature Normalization; KE - Kernel-based Feature Engineering; SRR - Smart Redundancy Reduction; SSYN - Sample Synthetizer.

| **Preprocessing step** | **Algorithm** | **Parameter** | **Value** | **Reference** |
| --- | --- | --- | --- | --- |
| 1 | FN | Normalization type | Mean-Deviation | (4) |
| 2 | KE | Kernels applied | Gaussian; Polynomial; Tanh | (5) |
| 3 | FN | Normalization type | Mean-Deviation | (4) |
| 4 | SRR | Redundancy Threshold (Covariance) | 0.85 | (6) |
| 5 | SSYN | Oversampling ratio (majority subgroup) | 1.25; 1.5; 1.62; 1.75; 1.87 | (7) |
|  |  | Sampling technique | SMOTE |  |

**Machine Learning Layer 1**

Various machine learning algorithms were established in each fold to minimize the effect of algorithm bias (6). Each model was trained by randomly selecting 80% of the preprocessed training data per MC fold. For details of the ML algorithms, see Supplementary Table 9.

**Supplementary Table 9.** Machine learning (ML) algorithms in the first ML layer with their parameters and value ranges across Monte Carlo (MC) folds. Occurrence of each ML type is represented in percentages across MC folds. MGWC – Multi-Gaussian Weighted Classifier; RF – Random Forest Classifier; SVM – Support Vector Machine Classifier;

| **ML Algorithm** | **Parameter** | **Value Range** | **Occurrence** | **Reference** |
| --- | --- | --- | --- | --- |
| MGWC | Initial value multiplier | 1 – 10 | 35.93% | (8) |
|  | Maximum iterations | 10000 – 75000 |  |  |
|  | Negative weights allowed | false, true |  |  |
|  | Scale value multiplier | 0.1 – 50 |  |  |
|  | Tolerance | 0.00001 – 0.0001 |  |  |
| RF | Bag fraction | 0.8 – 0.99 | 29.84% | (6) |
|  | Bagging method | equalized, normal |  |  |
|  | Boosting | none, adaboost |  |  |
|  | Maximum tree depth | 5 – 10 |  |  |
|  | Minimum samples in leaves | 2 – 5 |  |  |
|  | Node feature selection method | none |  |  |
|  | Number of random features per node | 5 |  |  |
|  | Number of selected trees | 101 – 201 |  |  |
|  | Number of trees to build | 301 – 1001 |  |  |
|  | Tree quality metric | gain, gini |  |  |
|  | Tree selection method | 0 |  |  |
| SVM | Learning rate | 0.001 – 0.01 | 34.22% | (9) |
|  | Maximum iterations | 1000 – 5000 |  |  |

**Machine Learning Layer 2**

Meta-training sets were created by evaluating the samples of the preprocessed training set in each MC fold by the trained models in ML layer 1. In order to create the meta-training set, the prediction results of each trained model in ML layer 1 were handled as feature values of the given training sample. The meta-training set was the input for training the second ML layer prediction models. These models were trained to identify patterns in the prediction of the first ML layer models to result in mixed super learners (10). For the parameters of the second layer ML algorithms see Supplementary Table 10.

**Supplementary Table 10.** Machine learning (ML) algorithms in the second ML layer with their parameters and value ranges across Monte Carlo (MC) folds. Occurrence of each ML type is represented in percentages across MC folds. MGWC – Multi-Gaussian Weighted Classifier; RF – Random Forest Classifier; SVM – Support Vector Machine Classifier;

| **ML Algorithm** | **Parameter** | **Value Range** | **Occurrence** | **Reference** |
| --- | --- | --- | --- | --- |
| MGWC | Initial value multiplier | 10 | 33.33% | (8) |
|  | Maximum iterations | 3000 – 13000 |  |  |
|  | Negative weights allowed | false, true |  |  |
|  | Scale value multiplier | 1 – 5 |  |  |
|  | Tolerance | 0.0001 |  |  |
| RF | Bag fraction | 0.8 – 0.99 | 33.33% | (6) |
|  | Bagging method | equalized, normal |  |  |
|  | Boosting | none, adaboost |  |  |
|  | Maximum tree depth | 5 |  |  |
|  | Minimum samples in leaves | 2 – 5 |  |  |
|  | Node feature selection method | none |  |  |
|  | Number of random features per node | 5 |  |  |
|  | Number of selected trees | 201 |  |  |
|  | Number of trees to build | 501 – 1001 |  |  |
|  | Tree quality metric | gain, gini |  |  |
|  | Tree selection method | 0 |  |  |
| SVM | Learning rate | 0.001 – 0.01 | 33.33% | (9) |
|  | Maximum iterations | 1000 – 5000 |  |  |

**Top-Layer Model**

Combination of the prediction results of the second layer ML models was performed by weighted majority voting to provide the final prediction of the model scheme. Weighting of each ML Layer 2 model was calculated based on training performance. In addition, ML Layer 2 models having less training performance than the median of all ML layer 2 model training performances had weight 0 in the final vote.

**RESULTS**

**Cross-Validation Performance**

Model prediction performance was estimated via the MC cross-validation scheme utilizing confusion matrix analytics (11). True positive, true negative, false positive and false negative confusion matrix entries were calculated by evaluating the validation samples by the established model pipeline in each fold. Sensitivity, specificity, accuracy, positive predictive as well as negative predictive values were calculated across the MC fold validation results. For the average cross-validation performance of ML Layer 1 and 2 models see Supplementary Table 11 and Supplementary Table 12 respectively. For the cross-validation results of the final (top-layer) prediction models as well as for the summary of the evaluation, see Supplementary Table 13.

**Supplementary Table 11. Average Monte Carlo (MC) cross-validation performance (%) of ML Layer 1 (ML-1) predictive models as determined by confusion matrix analytics across all MC folds.** MGWC – Multi-Gaussian Weighted Classifier; RF – Random Forest Classifier; SVM – Support Vector Machine Classifier; SNS – Sensitivity; SPC – Specificity; PPV – Positive Predictive Value; NPV – Negative Predictive Value; ACC – Accuracy; OCC – Occurrence. Performance and occurrence values are in percentages.

|  | **SNS** | **SPC** | **PPV** | **NPV** | **ACC** | **OCC** |
| --- | --- | --- | --- | --- | --- | --- |
| **MGWC** | 73 | 71 | 73 | 74 | 72 | 36 |
| **RF** | 66 | 70 | 70 | 70 | 68 | 30 |
| **SVM** | 74 | 73 | 75 | 76 | 74 | 34 |

**Supplementary Table 12. Average Monte Carlo (MC) cross-validation performance (%) of ML Layer 2 (ML-2) predictive models as determined by confusion matrix analytics across all MC folds.** MGWC – Multi-Gaussian Weighted Classifier; RF – Random Forest Classifier; SVM – Support Vector Machine Classifier; SNS – Sensitivity; SPC – Specificity; PPV – Positive Predictive Value; NPV – Negative Predictive Value; ACC – Accuracy; OCC – Occurrence. Performance and occurrence values are in percentages.

|  | **SNS** | **SPC** | **PPV** | **NPV** | **ACC** | **OCC** |
| --- | --- | --- | --- | --- | --- | --- |
| **MGWC** | 74 | 72 | 75 | 76 | 73 | 33 |
| **RF** | 66 | 69 | 70 | 69 | 67 | 33 |
| **SVM** | 76 | 74 | 75 | 77 | 75 | 33 |

**Supplementary Table 13.** Performance Monte Carlo (MC) cross-validation performance of the established model scheme throughout the performance of the top-layer prediction model. Performance values were determined by confusion matrix analytics across all MC folds. MGWC – Multi-Gaussian Weighted Classifier; RF – Random Forest Classifier; SVM – Support Vector Machine Classifier; SNS – Sensitivity; SPC – Specificity; PPV – Positive Predictive Value; NPV – Negative Predictive Value; ACC – Accuracy; AUC – Area Under the Receiver Operator Characteristics Curve. Performance values are in percentages. LQ – Lower quartile; UQ – Upper Quartile; Dev – Deviation.

|  | **Min** | **LQ** | **Median** | **UQ** | **Max** | **Mean** | **Dev** |
| --- | --- | --- | --- | --- | --- | --- | --- |
| **SNS** | 16.66 | 66.66 | 66.66 | 83.33 | 100 | 70.66 | 15.89 |
| **SPC** | 16.66 | 66.66 | 66.66 | 83.33 | 100 | 70.83 | 15.91 |
| **PPV** | 25 | 60 | 71.42 | 81.66 | 100 | 72.16 | 12.84 |
| **NPV** | 33.33 | 62.5 | 71.42 | 83.33 | 100 | 72.38 | 12.92 |
| **ACC** | 33.33 | 62.5 | 75 | 83.33 | 100 | 70.75 | 11.33 |
| **AUC** | 37.5 | 65.27 | 73.61 | 83.33 | 100 | 73.48 | 10.71 |

**Feature Importance**

Feature ranking and selection was performed as part of the data preprocessing steps of each fold (see Sec. Preprocessing). The final feature importance was calculated as the mean of all feature rankings across the MC folds.

**Supplementary Table 14.** Selected features and their ranks as calculated across the MC folds by Smart Redundancy Reduction (SRR - see Supplementary Table 8) as well as their respective value distributions. Ranks represent the relative importance of selected features for model building. Features are ordered by ranks. Rank values are in percentages.

| **Feature Name** | **Ranking** | **Histogram** |
| --- | --- | --- |
| Cellular senescence-CT::Histog  ram::ih.kurt-0-1 | 9.91% |  |
| Cellular senescence-CT::GLCM::  cm.clust.shade-1-1 | 8.99% |  |
| Cellular senescence-CT::GLSZM:  :szm.lzhge-1-0 | 7.6% |  |
| p53 signaling pathway-CT::GLSZ  M::szm.lzhge-1-0 | 6.77% |  |
| Apoptosis-CT::GLSZM::szm.z.per  c-1-1 | 6.3% |  |
| Cellular senescence-CT::GLSZM:  :szm.z.perc-0-0 | 5.92% |  |
| Cellular senescence-CT::GLSZM:  :szm.z.perc-1-1 | 5.86% |  |
| p53 signaling pathway-CT::Hist  ogram::ih.kurt-0-1 | 5.75% |  |
| Apoptosis-CT::GLSZM::szm.lzhge  -1-0 | 5.68% |  |
| Cellular senescence-PET::Inten  sity::stat.sum-1-0 | 5.1% |  |
| Cellular senescence | 4.53% |  |
| Cellular senescence-CT::Morpho  logical::morph.vol-1-0 | 4.26% |  |
| CT::GLSZM::szm.z.perc | 4.02% |  |
| Apoptosis-CT::Morphological::m  orph.vol-1-0 | 3.68% |  |
| Apoptosis-PET::Intensity::stat  .sum-1-0 | 3.41% |  |
| Apoptosis | 3.31% |  |
| CT::GLSZM::szm.lzhge | 2.79% |  |
| p53 signaling pathway-CT::GLSZ  M::szm.z.perc-0-0 | 2.43% |  |
| CT::Morphological::morph.vol | 1.98% |  |
| PET::Intensity::stat.sum | 1.62% |  |

Machine learning analysis for the median-OS classification model

**AUTOMATED MACHINE LEARNING ANALYSIS**

Tabular data submission for Dedicaid AutoML services was performed by Dedicaid user clemens.spielvogel@meduniwien.ac.at on 8/1/2021, 3:58:46 PM to build and cross-validate automated data preprocessing and mixed, stacked ensemble learning pipelines for predicting reference label OS_histo_median. For the details of the analysis see Supplementary Table 15.

**Supplementary Table 15.** Properties of the automated machine learning (AutoML) analysis of this study.

| Data name | fdb_multiomics_w_labels_bonferroni_significant.xlsx |
| --- | --- |
| Data size | 62 samples, 20 features |
| Date of analysis | 8/1/2021, 3:58:46 PM |
| Duration of analysis | 3h 31s |
| Submitted by | clemens.spielvogel@meduniwien.ac.at |
| Dedicaid AutoML version | 0.1 |

**METHODS**

**Data**

The input dataset was composed of 62 samples and 23 features. The submitted dataset was composed of 20 features. The selected reference label for the cross-validation was OS_histo_median having subgroups of 0 (50%) and 1 (50%) label outcomes.

**Cross-Validation**

Monte Carlo (MC) cross-validation scheme was applied with 80% training and 20% validation ratios across 100 folds (3). Each fold had unique training-validation configurations. MC split resulted in 50 samples per fold in the training set. The validation set of each fold contained 6 samples per reference label (12 overall). The validation samples were equally subsampled to ensure that none of the label outcomes are over or underrepresented during the cross-validation.

**Preprocessing**

The data underwent preprocessing steps in each fold before performing machine learning (ML) analysis. Preprocessing resulted in average 84 samples and 14 features across all MC folds. For the preprocessing steps and their parameters, see Supplementary Table 16.

**Supplementary Table 16.** Preprocessing step algorithms as well as their parameter values performed in all Monte Carlo folds before machine learning. FN - Feature Normalization; KE - Kernel-based Feature Engineering; SRR - Smart Redundancy Reduction; SSYN - Sample Synthetizer.

| **Preprocessing step** | **Algorithm** | **Parameter** | **Value** | **Reference** |
| --- | --- | --- | --- | --- |
| 1 | FN | Normalization type | Mean-Deviation | (4) |
| 2 | KE | Kernels applied | Gaussian; Polynomial; Tanh | (5) |
| 3 | FN | Normalization type | Mean-Deviation | (4) |
| 4 | SRR | Redundancy Threshold (Covariance) | 0.85 | (6) |
| 5 | SSYN | Oversampling ratio (majority subgroup) | 1.25; 1.5; 1.75 | (7) |
|  |  | Sampling technique | SMOTE |  |

**Machine Learning Layer 1**

Various machine learning algorithms were established in each fold to minimize the effect of algorithm bias (6). Each model was trained by randomly selecting 80% of the preprocessed training data per MC fold. For details of the ML algorithms, see Supplementary Table 17.

**Supplementary Table 17.** Machine learning (ML) algorithms in the first ML layer with their parameters and value ranges across Monte Carlo (MC) folds. Occurrence of each ML type is represented in percentages across MC folds. MGWC – Multi-Gaussian Weighted Classifier; RF – Random Forest Classifier; SVM – Support Vector Machine Classifier;

| **ML Algorithm** | **Parameter** | **Value Range** | **Occurrence** | **Reference** |
| --- | --- | --- | --- | --- |
| MGWC | Initial value multiplier | 1 – 10 | 32.26% | (8) |
|  | Maximum iterations | 8000 – 70000 |  |  |
|  | Negative weights allowed | false, true |  |  |
|  | Scale value multiplier | 0.1 – 50 |  |  |
|  | Tolerance | 0.00001 – 0.0001 |  |  |
| RF | Bag fraction | 0.8 – 0.99 | 31.89% | (6) |
|  | Bagging method | equalized, normal |  |  |
|  | Boosting | none, adaboost |  |  |
|  | Maximum tree depth | 5 – 10 |  |  |
|  | Minimum samples in leaves | 2 – 5 |  |  |
|  | Node feature selection method | none |  |  |
|  | Number of random features per node | 5 |  |  |
|  | Number of selected trees | 101 – 201 |  |  |
|  | Number of trees to build | 301 – 1001 |  |  |
|  | Tree quality metric | gain, gini |  |  |
|  | Tree selection method | 0 |  |  |
| SVM | Learning rate | 0.001 – 0.01 | 35.83% | (9) |
|  | Maximum iterations | 1000 – 5000 |  |  |

**Machine Learning Layer 2**

Meta-training sets were created by evaluating the samples of the preprocessed training set in each MC fold by the trained models in ML layer 1. In order to create the meta-training set, the prediction results of each trained model in ML layer 1 were handled as feature values of the given training sample. The meta-training set was the input for training the second ML layer prediction models. These models were trained to identify patterns in the prediction of the first ML layer models to result in mixed super learners (10). For the parameters of the second layer ML algorithms see Supplementary Table 18.

**Supplementary Table 18.** Machine learning (ML) algorithms in the second ML layer with their parameters and value ranges across Monte Carlo (MC) folds. Occurrence of each ML type is represented in percentages across MC folds. MGWC – Multi-Gaussian Weighted Classifier; RF – Random Forest Classifier; SVM – Support Vector Machine Classifier;

| **ML Algorithm** | **Parameter** | **Value Range** | **Occurrence** | **Reference** |
| --- | --- | --- | --- | --- |
| MGWC | Initial value multiplier | 10 | 33.33% | (8) |
|  | Maximum iterations | 3000 – 14000 |  |  |
|  | Negative weights allowed | false, true |  |  |
|  | Scale value multiplier | 1 – 5 |  |  |
|  | Tolerance | 0.0001 |  |  |
| RF | Bag fraction | 0.8 – 0.99 | 33.33% | (6) |
|  | Bagging method | equalized, normal |  |  |
|  | Boosting | none, adaboost |  |  |
|  | Maximum tree depth | 5 |  |  |
|  | Minimum samples in leaves | 2 – 5 |  |  |
|  | Node feature selection method | none |  |  |
|  | Number of random features per node | 5 |  |  |
|  | Number of selected trees | 201 |  |  |
|  | Number of trees to build | 501 – 1001 |  |  |
|  | Tree quality metric | gain, gini |  |  |
|  | Tree selection method | 0 |  |  |
| SVM | Learning rate | 0.001 – 0.01 | 33.33% | (9) |
|  | Maximum iterations | 1000 – 5000 |  |  |

**Top-Layer Model**

Combination of the prediction results of the second layer ML models was performed by weighted majority voting to provide the final prediction of the model scheme. Weighting of each ML Layer 2 model was calculated based on training performance. In addition, ML Layer 2 models having less training performance than the median of all ML layer 2 model training performances had weight 0 in the final vote.

**RESULTS**

**Cross-Validation Performance**

Model prediction performance was estimated via the MC cross-validation scheme utilizing confusion matrix analytics (11). True positive, true negative, false positive and false negative confusion matrix entries were calculated by evaluating the validation samples by the established model pipeline in each fold. Sensitivity, specificity, accuracy, positive predictive as well as negative predictive values were calculated across the MC fold validation results. For the average cross-validation performance of ML Layer 1 and 2 models see Supplementary Table 19 and Supplementary Table 20 respectively. For the cross-validation results of the final (top-layer) prediction models as well as for the summary of the evaluation, see Supplementary Table 21.

**Supplementary Table 19.** Average Monte Carlo (MC) cross-validation performance (%) of ML Layer 1 (ML-1) predictive models as determined by confusion matrix analytics across all MC folds. MGWC – Multi-Gaussian Weighted Classifier; RF – Random Forest Classifier; SVM – Support Vector Machine Classifier; SNS – Sensitivity; SPC – Specificity; PPV – Positive Predictive Value; NPV – Negative Predictive Value; ACC – Accuracy; OCC – Occurrence. Performance and occurrence values are in percentages.

|  | **SNS** | **SPC** | **PPV** | **NPV** | **ACC** | **OCC** |
| --- | --- | --- | --- | --- | --- | --- |
| **MGWC** | 74 | 72 | 74 | 76 | 73 | 32 |
| **RF** | 63 | 72 | 70 | 69 | 67 | 32 |
| **SVM** | 76 | 73 | 76 | 77 | 75 | 36 |

**Supplementary** **Table 20.** Average Monte Carlo (MC) cross-validation performance (%) of ML Layer 2 (ML-2) predictive models as determined by confusion matrix analytics across all MC folds. MGWC – Multi-Gaussian Weighted Classifier; RF – Random Forest Classifier; SVM – Support Vector Machine Classifier; SNS – Sensitivity; SPC – Specificity; PPV – Positive Predictive Value; NPV – Negative Predictive Value; ACC – Accuracy; OCC – Occurrence. Performance and occurrence values are in percentages.

|  | **SNS** | **SPC** | **PPV** | **NPV** | **ACC** | **OCC** |
| --- | --- | --- | --- | --- | --- | --- |
| **MGWC** | 75 | 74 | 76 | 77 | 74 | 33 |
| **RF** | 64 | 69 | 69 | 67 | 66 | 33 |
| **SVM** | 76 | 74 | 77 | 78 | 75 | 33 |

**Supplementary Table 21.** Performance Monte Carlo (MC) cross-validation performance of the established model scheme throughout the performance of the top-layer prediction model. Performance values were determined by confusion matrix analytics across all MC folds. MGWC – Multi-Gaussian Weighted Classifier; RF – Random Forest Classifier; SVM – Support Vector Machine Classifier; SNS – Sensitivity; SPC – Specificity; PPV – Positive Predictive Value; NPV – Negative Predictive Value; ACC – Accuracy; AUC – Area Under the Receiver Operator Characteristics Curve. Performance values are in percentages. LQ – Lower quartile; UQ – Upper Quartile; Dev – Deviation.

|  | **Min** | **LQ** | **Median** | **UQ** | **Max** | **Mean** | **Dev** |
| --- | --- | --- | --- | --- | --- | --- | --- |
| **SNS** | 16.66 | 58.33 | 66.66 | 83.33 | 100 | 69.66 | 15.05 |
| **SPC** | 33.33 | 66.66 | 66.66 | 83.33 | 100 | 72.16 | 14.27 |
| **PPV** | 40 | 62.5 | 75 | 83.33 | 100 | 73.46 | 11.58 |
| **NPV** | 40 | 61.25 | 71.42 | 83.33 | 100 | 72.39 | 11.68 |
| **ACC** | 41.66 | 66.66 | 75 | 83.33 | 100 | 70.91 | 9.49 |
| **AUC** | 41.66 | 66.66 | 73.61 | 81.94 | 100 | 73.54 | 8.93 |

**Feature Importance**

Feature ranking and selection was performed as part of the data preprocessing steps of each fold (see Sec. Preprocessing). The final feature importance was calculated as the mean of all feature rankings across the MC folds.

**Supplementary Table 22.** Selected features and their ranks as calculated across the MC folds by Smart Redundancy Reduction (SRR - see Supplementary Table 16) as well as their respective value distributions. Ranks represent the relative importance of selected features for model building. Features are ordered by ranks. Rank values are in percentages.

| **Feature Name** | **Ranking** | **Histogram** |
| --- | --- | --- |
| Cellular senescence-CT::Histog  ram::ih.kurt-0-1 | 9.68% |  |
| Cellular senescence-CT::GLCM::  cm.clust.shade-1-1 | 8.76% |  |
| Cellular senescence-CT::GLSZM:  :szm.lzhge-1-0 | 7.66% |  |
| p53 signaling pathway-CT::GLSZ  M::szm.lzhge-1-0 | 6.6% |  |
| Apoptosis-CT::GLSZM::szm.z.per  c-1-1 | 6.37% |  |
| Cellular senescence-CT::GLSZM:  :szm.z.perc-1-1 | 5.86% |  |
| Cellular senescence-CT::GLSZM:  :szm.z.perc-0-0 | 5.82% |  |
| p53 signaling pathway-CT::Hist  ogram::ih.kurt-0-1 | 5.79% |  |
| Cellular senescence-PET::Inten  sity::stat.sum-1-0 | 5.5% |  |
| Apoptosis-CT::GLSZM::szm.lzhge  -1-0 | 5.06% |  |
| Cellular senescence | 4.44% |  |
| Apoptosis-CT::Morphological::m  orph.vol-1-0 | 4.4% |  |
| CT::GLSZM::szm.z.perc | 4.05% |  |
| Cellular senescence-CT::Morpho  logical::morph.vol-1-0 | 3.87% |  |
| Apoptosis-PET::Intensity::stat  .sum-1-0 | 3.43% |  |
| Apoptosis | 3.38% |  |
| CT::GLSZM::szm.lzhge | 2.8% |  |
| p53 signaling pathway-CT::GLSZ  M::szm.z.perc-0-0 | 2.67% |  |
| CT::Morphological::morph.vol | 2.42% |  |
| PET::Intensity::stat.sum | 1.34% |  |

Machine learning analysis for the 36-months-OS classification model

**AUTOMATED MACHINE LEARNING ANALYSIS**

Tabular data submission for Dedicaid AutoML services was performed by Dedicaid user clemens.spielvogel@meduniwien.ac.at on 8/1/2021, 3:59:04 PM to build and cross-validate automated data preprocessing and mixed, stacked ensemble learning pipelines for predicting reference label OS_histo36. For the details of the analysis see Supplementary Table 23.

**Supplementary Table 23.** Properties of the automated machine learning (AutoML) analysis of this study.

| Data name | fdb_multiomics_w_labels_bonferroni_significant.xlsx |
| --- | --- |
| Data size | 62 samples, 20 features |
| Date of analysis | 8/1/2021, 3:59:04 PM |
| Duration of analysis | 6h 4m 7s |
| Submitted by | clemens.spielvogel@meduniwien.ac.at |
| Dedicaid AutoML version | 0.1 |

**METHODS**

**Data**

The input dataset was composed of 62 samples and 23 features. The submitted dataset was composed of 20 features. The selected reference label for the cross-validation was OS_histo36 having subgroups of 0 (61.2%) and 1 (38.7%) label outcomes.

**Cross-Validation**

Monte Carlo (MC) cross-validation scheme was applied with 80% training and 20% validation ratios across 100 folds (3). Each fold had unique training-validation configurations. MC split resulted in 50 samples per fold in the training set. The validation set of each fold contained 6 samples per reference label (12 overall). The validation samples were equally subsampled to ensure that none of the label outcomes are over or underrepresented during the cross-validation.

**Preprocessing**

The data underwent preprocessing steps in each fold before performing machine learning (ML) analysis. Preprocessing resulted in average 125 samples and 16 features across all MC folds. For the preprocessing steps and their parameters, see Supplementary Table 24.

**Supplementary Table 24.** Preprocessing step algorithms as well as their parameter values performed in all Monte Carlo folds before machine learning. FN - Feature Normalization; KE - Kernel-based Feature Engineering; SRR - Smart Redundancy Reduction; SSYN - Sample Synthetizer.

| **Preprocessing step** | **Algorithm** | **Parameter** | **Value** | **Reference** |
| --- | --- | --- | --- | --- |
| 1 | FN | Normalization type | Mean-Deviation | (4) |
| 2 | KE | Kernels applied | Gaussian; Polynomial; Tanh | (5) |
| 3 | FN | Normalization type | Mean-Deviation | (4) |
| 4 | SRR | Redundancy Threshold (Covariance) | 0.85 | (6) |
| 5 | SSYN | Oversampling ratio (majority subgroup) | 1.62; 1.86 | (7) |
|  |  | Sampling technique | SMOTE |  |

**Machine Learning Layer 1**

Various machine learning algorithms were established in each fold to minimize the effect of algorithm bias (6). Each model was trained by randomly selecting 80% of the preprocessed training data per MC fold. For details of the ML algorithms, see Supplementary Table 25.

**Supplementary Table 25.** Machine learning (ML) algorithms in the first ML layer with their parameters and value ranges across Monte Carlo (MC) folds. Occurrence of each ML type is represented in percentages across MC folds. MGWC – Multi-Gaussian Weighted Classifier; RF – Random Forest Classifier; SVM – Support Vector Machine Classifier;

| **ML Algorithm** | **Parameter** | **Value Range** | **Occurrence** | **Reference** |
| --- | --- | --- | --- | --- |
| MGWC | Initial value multiplier | 1 – 10 | 34.27% | (8) |
|  | Maximum iterations | 14000 – 80000 |  |  |
|  | Negative weights allowed | true, false |  |  |
|  | Scale value multiplier | 0.1 – 50 |  |  |
|  | Tolerance | 0.00001 – 0.0001 |  |  |
| RF | Bag fraction | 0.8 – 0.99 | 32.34% | (6) |
|  | Bagging method | normal, equalized |  |  |
|  | Boosting | adaboost, none |  |  |
|  | Maximum tree depth | 5 – 10 |  |  |
|  | Minimum samples in leaves | 2 – 5 |  |  |
|  | Node feature selection method | none |  |  |
|  | Number of random features per node | 5 |  |  |
|  | Number of selected trees | 101 – 201 |  |  |
|  | Number of trees to build | 301 – 1001 |  |  |
|  | Tree quality metric | gini, gain |  |  |
|  | Tree selection method | 0 |  |  |
| SVM | Learning rate | 0.001 – 0.01 | 33.37% | (9) |
|  | Maximum iterations | 1000 – 5000 |  |  |

**Machine Learning Layer 2**

Meta-training sets were created by evaluating the samples of the preprocessed training set in each MC fold by the trained models in ML layer 1. In order to create the meta-training set, the prediction results of each trained model in ML layer 1 were handled as feature values of the given training sample. The meta-training set was the input for training the second ML layer prediction models. These models were trained to identify patterns in the prediction of the first ML layer models to result in mixed super learners (10). For the parameters of the second layer ML algorithms see Supplementary Table 26.

**Supplementary Table 26.** Machine learning (ML) algorithms in the second ML layer with their parameters and value ranges across Monte Carlo (MC) folds. Occurrence of each ML type is represented in percentages across MC folds. MGWC – Multi-Gaussian Weighted Classifier; RF – Random Forest Classifier; SVM – Support Vector Machine Classifier;

| **ML Algorithm** | **Parameter** | **Value Range** | **Occurrence** | **Reference** |
| --- | --- | --- | --- | --- |
| MGWC | Initial value multiplier | 10 | 33.33% | (8) |
|  | Maximum iterations | 4000 – 12000 |  |  |
|  | Negative weights allowed | true, false |  |  |
|  | Scale value multiplier | 1 – 5 |  |  |
|  | Tolerance | 0.0001 |  |  |
| RF | Bag fraction | 0.8 – 0.99 | 33.33% | (6) |
|  | Bagging method | normal, equalized |  |  |
|  | Boosting | none, adaboost |  |  |
|  | Maximum tree depth | 5 |  |  |
|  | Minimum samples in leaves | 2 – 5 |  |  |
|  | Node feature selection method | none |  |  |
|  | Number of random features per node | 5 |  |  |
|  | Number of selected trees | 201 |  |  |
|  | Number of trees to build | 501 – 1001 |  |  |
|  | Tree quality metric | gini, gain |  |  |
|  | Tree selection method | 0 |  |  |
| SVM | Learning rate | 0.001 – 0.01 | 33.33% | (9) |
|  | Maximum iterations | 1000 – 5000 |  |  |

**Top-Layer Model**

Combination of the prediction results of the second layer ML models was performed by weighted majority voting to provide the final prediction of the model scheme. Weighting of each ML Layer 2 model was calculated based on training performance. In addition, ML Layer 2 models having less training performance than the median of all ML layer 2 model training performances had weight 0 in the final vote.

**RESULTS**

**Cross-Validation Performance**

Model prediction performance was estimated via the MC cross-validation scheme utilizing confusion matrix analytics (11). True positive, true negative, false positive and false negative confusion matrix entries were calculated by evaluating the validation samples by the established model pipeline in each fold. Sensitivity, specificity, accuracy, positive predictive as well as negative predictive values were calculated across the MC fold validation results. For the average cross-validation performance of ML Layer 1 and 2 models see Supplementary Table 27 and Supplementary Table 28 respectively. For the cross-validation results of the final (top-layer) prediction models as well as for the summary of the evaluation, see Supplementary Table 29.

**Supplementary Table 27.** Average Monte Carlo (MC) cross-validation performance (%) of ML Layer 1 (ML-1) predictive models as determined by confusion matrix analytics across all MC folds. MGWC – Multi-Gaussian Weighted Classifier; RF – Random Forest Classifier; SVM – Support Vector Machine Classifier; SNS – Sensitivity; SPC – Specificity; PPV – Positive Predictive Value; NPV – Negative Predictive Value; ACC – Accuracy; OCC – Occurrence. Performance and occurrence values are in percentages.

|  | **SNS** | **SPC** | **PPV** | **NPV** | **ACC** | **OCC** |
| --- | --- | --- | --- | --- | --- | --- |
| **MGWC** | 74 | 75 | 78 | 78 | 75 | 34 |
| **RF** | 57 | 79 | 73 | 67 | 68 | 32 |
| **SVM** | 87 | 67 | 74 | 86 | 77 | 33 |

**Supplementary Table 28.** Average Monte Carlo (MC) cross-validation performance (%) of ML Layer 2 (ML-2) predictive models as determined by confusion matrix analytics across all MC folds. MGWC – Multi-Gaussian Weighted Classifier; RF – Random Forest Classifier; SVM – Support Vector Machine Classifier; SNS – Sensitivity; SPC – Specificity; PPV – Positive Predictive Value; NPV – Negative Predictive Value; ACC – Accuracy; OCC – Occurrence. Performance and occurrence values are in percentages.

|  | **SNS** | **SPC** | **PPV** | **NPV** | **ACC** | **OCC** |
| --- | --- | --- | --- | --- | --- | --- |
| **MGWC** | 80 | 73 | 76 | 82 | 76 | 33 |
| **RF** | 58 | 77 | 72 | 68 | 67 | 33 |
| **SVM** | 86 | 68 | 75 | 86 | 77 | 33 |

**Supplementary Table 29.** Performance Monte Carlo (MC) cross-validation performance of the established model scheme throughout the performance of the top-layer prediction model. Performance values were determined by confusion matrix analytics across all MC folds. MGWC – Multi-Gaussian Weighted Classifier; RF – Random Forest Classifier; SVM – Support Vector Machine Classifier; SNS – Sensitivity; SPC – Specificity; PPV – Positive Predictive Value; NPV – Negative Predictive Value; ACC – Accuracy; AUC – Area Under the Receiver Operator Characteristics Curve. Performance values are in percentages. LQ – Lower quartile; UQ – Upper Quartile; Dev – Deviation.

|  | **Min** | **LQ** | **Median** | **UQ** | **Max** | **Mean** | **Dev** |
| --- | --- | --- | --- | --- | --- | --- | --- |
| **SNS** | 0 | 50 | 62.5 | 75 | 100 | 61 | 23 |
| **SPC** | 25 | 62.5 | 75 | 100 | 100 | 75.25 | 13.86 |
| **PPV** | 0 | 60 | 75 | 100 | 100 | 70.98 | 16.54 |
| **NPV** | 33.33 | 60 | 66.66 | 75 | 100 | 69.73 | 13.92 |
| **ACC** | 37.5 | 62.5 | 75 | 75 | 100 | 68.12 | 11.53 |
| **AUC** | 37.5 | 65.62 | 75 | 87.5 | 100 | 76.12 | 11.03 |

**Feature Importance**

Feature ranking and selection was performed as part of the data preprocessing steps of each fold (see Sec. Preprocessing). The final feature importance was calculated as the mean of all feature rankings across the MC folds.

**Supplementary Table 30.** Selected features and their ranks as calculated across the MC folds by Smart Redundancy Reduction (SRR - see Supplementary Table 24) as well as their respective value distributions. Ranks represent the relative importance of selected features for model building. Features are ordered by ranks. Rank values are in percentages.

| **Feature Name** | **Ranking** | **Histogram** |
| --- | --- | --- |
| Cellular senescence-CT::Histog  ram::ih.kurt-0-1 | 10.03% |  |
| Cellular senescence-CT::GLSZM:  :szm.lzhge-1-0 | 7.39% |  |
| Apoptosis-PET::Intensity::stat  .sum-1-0 | 7.05% |  |
| Cellular senescence-PET::Inten  sity::stat.sum-1-0 | 7.04% |  |
| Cellular senescence-CT::GLCM::  cm.clust.shade-1-1 | 6.94% |  |
| Apoptosis-CT::GLSZM::szm.lzhge  -1-0 | 6.85% |  |
| Apoptosis-CT::GLSZM::szm.z.per  c-1-1 | 6.52% |  |
| Cellular senescence-CT::GLSZM:  :szm.z.perc-1-1 | 5.55% |  |
| Apoptosis | 4.99% |  |
| p53 signaling pathway-CT::GLSZ  M::szm.lzhge-1-0 | 4.86% |  |
| p53 signaling pathway-CT::Hist  ogram::ih.kurt-0-1 | 4.86% |  |
| Cellular senescence-CT::GLSZM:  :szm.z.perc-0-0 | 4.54% |  |
| Cellular senescence | 4.41% |  |
| Apoptosis-CT::Morphological::m  orph.vol-1-0 | 4.2% |  |
| Cellular senescence-CT::Morpho  logical::morph.vol-1-0 | 4% |  |
| CT::GLSZM::szm.z.perc | 2.93% |  |
| p53 signaling pathway-CT::GLSZ  M::szm.z.perc-0-0 | 2.62% |  |
| CT::GLSZM::szm.lzhge | 2.13% |  |
| CT::Morphological::morph.vol | 1.62% |  |
| PET::Intensity::stat.sum | 1.38% |  |

**References**

1. Arita H, Kinoshita M, Kawaguchi A, Takahashi M, Narita Y, Terakawa Y, et al. Lesion location implemented magnetic resonance imaging radiomics for predicting IDH and TERT promoter mutations in grade II/III gliomas. Sci Rep [Internet]. 2018 Dec 6;8(1):11773. Available from: http://www.nature.com/articles/s41598-018-30273-4

2. van der Maaten LJP, Hinton GE. Visualizing High-Dimensional Data Using t-SNE. Journal of Machine Learning Research. 2008;9(nov):2579-2605. Available from: https://jmlr.org/papers/volume9/vandermaaten08a/vandermaaten08a.pdf

3. Papp L, Spielvogel CP, Rausch I, Hacker M, Beyer T. Personalizing Medicine Through Hybrid Imaging and Medical Big Data Analysis. Front Phys [Internet]. 2018 Jun 7;6. Available from: https://www.frontiersin.org/article/10.3389/fphy.2018.00051/full

4. Han J, Pei J, Kamber M. Data Mining: Concepts and Techniques [Internet]. Elsevier Science; 2011. (The Morgan Kaufmann Series in Data Management Systems). Available from: https://books.google.at/books?id=pQws07tdpjoC

5. Souza CR. Kernel Functions for Machine Learning Applications [Internet]. 2020. Available from: http://crsouza.blogspot.com/2010/03/kernel-functions-for-machine-learning.html

6. Papp L, Spielvogel CP, Grubmüller B, Grahovac M, Krajnc D, Ecsedi B, et al. Supervised machine learning enables non-invasive lesion characterization in primary prostate cancer with [68Ga]Ga-PSMA-11 PET/MRI. Eur J Nucl Med Mol Imaging [Internet]. 2020 Dec 19; Available from: http://link.springer.com/10.1007/s00259-020-05140-y

7. Amin A, Anwar S, Adnan A, Nawaz M, Howard N, Qadir J, et al. Comparing Oversampling Techniques to Handle the Class Imbalance Problem: A Customer Churn Prediction Case Study. IEEE Access. 2016;4(October):7940–57.

8. Papp L, Pötsch N, Grahovac M, Schmidbauer V, Woehrer A, Preusser M, et al. Glioma survival prediction with combined analysis of in vivo 11C-MET PET features, ex vivo features, and patient features by supervised machine learning. J Nucl Med. 2018;59(6):892–9.

9. Gao X, Chu C, Li Y, Lu P, Wang W, Liu W, et al. The method and efficacy of support vector machine classifiers based on texture features and multi-resolution histogram from18F-FDG PET-CT images for the evaluation of mediastinal lymph nodes in patients with lung cancer. Eur J Radiol [Internet]. 2015;84(2):312–7. Available from: http://dx.doi.org/10.1016/j.ejrad.2014.11.006

10. van der Laan MJ, Polley EC, Hubbard AE. Super Learner. Stat Appl Genet Mol Biol [Internet]. 2007 Jan 16;6(1). Available from: https://www.degruyter.com/view/j/sagmb.2007.6.issue-1/sagmb.2007.6.1.1309/sagmb.2007.6.1.1309.xml

11. Stehman S V. Selecting and interpreting measures of thematic classification accuracy. Remote Sens Environ [Internet]. 1997 Oct;62(1):77–89. Available from: https://linkinghub.elsevier.com/retrieve/pii/S0034425797000837
